# Tracking of Intentionally Inoculated Lactic Acid Bacteria Strains in Yogurt and Probiotic Powder

**DOI:** 10.3390/microorganisms8010005

**Published:** 2019-12-18

**Authors:** Anshul Sharma, Jasmine Kaur, Sulhee Lee, Young-Seo Park

**Affiliations:** 1 Department of Food Science and Biotechnology, Gachon University, Gyeonggi-do 13120, Korea; anshul.silb18@gmail.com (A.S.); jasskaur.0612@gmail.com (J.K.); sulhee2340@gmail.com (S.L.); 2 Department of Food and Nutrition, Gachon University, Gyeonggi-do 13120, Korea; 3 Faculty of Applied Sciences and Biotechnology, Shoolini University of Biotechnology and Management Sciences, Bajhol, Solan, Himachal Pradesh 173229, India; 4 Research Group of Healthcare, Korea Food Research Institute, Wanju 55365, Korea

**Keywords:** lactic acid bacteria, *Leuconostoc mesenteroides*, *Lactobacillus brevis*, *Lactobacillus plantarum*, tracking, RAPD-PCR, rep-PCR, housekeeping genes

## Abstract

The present work aimed at tracking intentionally inoculated lactic acid bacteria (LAB) strains in yogurt and probiotic powder. *Leuconostoc (Leu.) mesenteroides* (11251), *Lactobacillus (L.) brevis* (B151), and *Lactobacillus plantarum* (LB41^K^) strains were tracked in yogurt, and *L. plantarum* (LB41^P^) was tracked in a commercial probiotic powder. The yogurt was intentionally inoculated with the selected bacterial strains. Two types of yogurt with known and unknown bacterial pools were utilized. The standard 16S rRNA gene sequencing was used to evaluate the initial screening. The molecular typing tools, random amplified polymorphic DNA (RAPD), repetitive element palindromic PCR (rep-PCR), and comparative gene sequence analysis of selected housekeeping loci were used to track the inoculated dubious strains. Out of 30 random selections for each inoculation, the developed method identified seven (11251), nine (B151), and five (LB41^K^) colonies in the yogurt. The validation was performed by identifying 7 colonies (LB41^P^) out of 30 in the probiotic powder. The DNA banding profiles and the gene sequence alignments led to the identification of the correct inoculated strains. Overall, the study summarizes the use of molecular tools to identify the deliberately inoculated LAB strains. In conclusion, the proposed polyphasic approach effectively tracked the intentionally inoculated strains: *Leu. mesenteroides*, *L. brevis*, and *L. plantarum* (LB41^K^) in yogurt and *L. plantarum* (LB41^P^) in probiotic powder. The study demonstrates how to track industrially relevant misused LAB strains in marketable food products.

## 1. Introduction

For over thousands of years, the intentional addition of bacteria to commercial products as starters or as food additives has been a continuous practice across the globe. These days, consumers are attracted more to food products with high quality, safety, and viable bacterial additives for societal health benefits [1]. On the other hand, food producers are showing interest in novel and industrially productive bacterial strains [2]. Among the industrial workhorses, lactic acid bacteria (LAB) are well thought of as unique and harmless bacteria, and most of the strains have earned the ‘‘generally recognized as safe’’ (GRAS) status [3]. Furthermore, the growing wealth of scientific literature has addressed the several health-promoting activities of LAB, including their immunomodulatory, antidiabetic, antiobesity, antioxidant, and anticancer activities [4,5]. In this era of a competitive world, the unscrupulous use of beneficial bacteria cannot be repudiated, which raises concerns about the safety of industrially relevant microorganisms. Hence, there is a necessity to develop measures to expose the dubious strains, which could be used for the manufacturing of food products.

The accurate species-specific documentation of LAB is significant from technical and safety perspectives [6]. A recent review article has meticulously described the use of various methodologies for the tracking of microorganisms [7]. Analytical approaches such as phenotypic and genotypic analyses have been exploited to identify and characterize LAB from food products [8,9]. Phenotypic approaches include morphological and physiological analysis, biochemical depiction, and protein profiling. However, these methods have low discriminatory power, lack sensitivity, and lack reproducibility [9]. The genotypic characterization of LAB involves a plethora of tools; they offer various advantages, including accuracy, reproducibility, and are timesaving [10,11]. In the present study, we utilized sequencing-based approaches such as 16S rRNA and housekeeping gene sequence analyses, and polymerase chain reaction (PCR)-based methods such as the random amplification of polymorphic DNA (RAPD) and repetitive genomic element (rep)-PCR to assess the fingerprint profile of the intentionally inoculated LAB strains.

RAPD is a molecular tool based on the random amplification of DNA fragments using short arbitrary sequence primers that bind at different locations within the genome, thereby generating a fingerprint of the bacteria [12]. The advantages of this tool include fastness, requiring less amount of DNA, flexible primer choice, and cost-effectiveness [13]. In contrast, the rep-PCR technique works by amplifying non-coding repetitive elements of bacterial DNA using specific primers for subspecies classification and the strain level discrimination [14,15]. The technique offers many advantages, such as fastness, cost-effectiveness, and high discriminative power [16]. Furthermore, the evaluation of the constitutive (housekeeping) loci as classification markers holds great potential in the expansion of globally available molecular tools for the documentation of LAB [17].

The industrialized food units are mostly interested in LAB species of the following genera: *Leuconostoc*, *Lactobacillus*, *Oenococcus*, *Lactococcus*, *Pediococcus*, *Streptococcus*, and *Enterococcus* [18]. Among them, three industrially important species, namely *Leuconostoc (Leu.) mesenteroides*, *Lactobacillus (L.) brevis*, and *Lactobacillus plantarum*, have been selected to validate the tracking technology. *Leuconostoc* species are mostly found on vegetables and fruits, and play an important role in various food and industrial fermentations; they are responsible for the organoleptic properties [12,19]. *L. brevis* has been reported in plants, fermented foods, and beverages, and its strains are characterized as probiotics [20,21]. Recently, *L. brevis* strain was also reported for its oral probiotic properties [22]. Contrariwise, *L. brevis* strains have also been reported for the spoilage of beer [23]. *L. plantarum* is a versatile and heterogeneous bacterium found in fermented foods and feed products, as well as in the human gastrointestinal tract (GIT) [24]. It is used in the processing of sauerkraut and cheese, and the fermentation of wine and green olives [25]. Some *L. plantarum* strains also synthesize vitamins and induce the production of host immunomodulatory particles [26,27]. In this study, considering their relevance, we evaluated the presence of the three deliberately inoculated industrially important strains, namely, *Leu. mesenteroides*, *L. plantarum*, and *L. brevis* in commercial yogurt by using various molecular tools. Further validation was assessed by tracing *L. plantarum* strains from a commercial probiotic powder.

## 2. Materials and Methods

### 2.1. Bacterial Strains

The three LAB strains used in this study were *Leu. mesenteroides* 11251, *L. plantarum* LB41^K^, and *L. brevis* B151. The strains were procured from the Korean culture collection of probiotics (KCCP) and maintained on De Man Rogosa Sharpe (MRS; Difco, Sparks, MD, USA) medium at 37 °C under aerobic conditions for 24 h. For the quantification of selected strains, the viable colonies were counted from 50 µL (serially diluted to 10^−6^) cultures grown on MRS agar plates and expressed as colony-forming units (CFU) per mL.

### 2.2. Yogurt and Probiotic Powder

**Figure 1:**
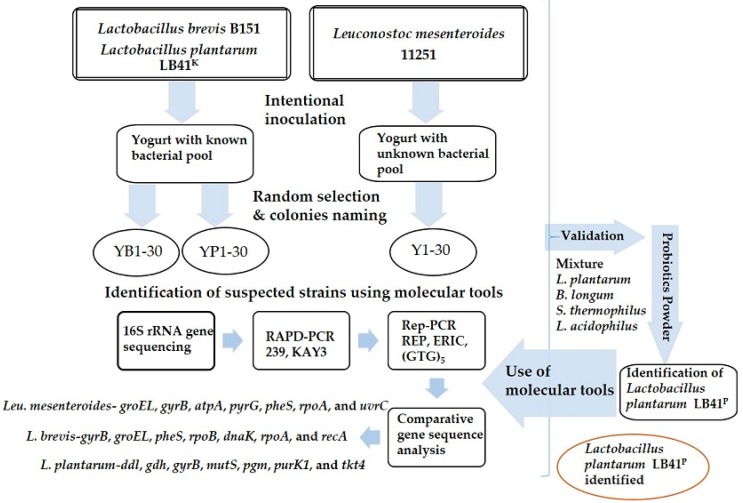
Flow chart of the presented technology. rep-PCR: repetitive element palindromic PCR, ERIC: enterobacterial repetitive intergenic consensus, RAPD: random amplified polymorphic DNA.

Two types of yogurts were purchased from a supermarket in Korea for a better insight of the presented technology to identify the suspected LAB strains. A yogurt with an unknown bacterial pool (Denmark drinking yogurt, Dongwon, Korea) was used for the deliberate addition of *Leu. mesenteroides*, whereas yogurts with known bacterial strains (*Streptococcus thermophilus*, *Lactobacillus casei*, *Bifidobacterium longum*, and *Lactobacillus acidophilus*; Mechnikop, Korea Yakult Co., Ltd., Seoul, Korea) were used for the inoculation of *L. brevis* and *L. plantarum*. The yogurts were inoculated (with a 10th of the total volume of the sample) using selected strains, rigorously vortexed, and serially diluted in phosphate-buffered saline (PBS; 10^−6^ dilution). The dilutions were spread plated on MRS agar plates and incubated for 24 h at 37 °C. The next day, cell viability was measured. For each experiment, 30 colonies were arbitrarily selected and labelled as Y1–30 for *Leu. mesenteroides*, YB1–30 for *L. brevis*, and YP1–30 for *L. plantarum*. For the final validation, a commercial probiotic powder (Aram Co., Inc., Gwangju, Korea) containing four bacterial strains (*L. plantarum*, *B. longum*, *S. thermophilus*, and *L. acidophilus*) was used to track the *L. plantarum* (LB41^P^) strain. The industry also provided the pure culture of the target strain (LB41^P^) as a reference. Figure 1 describes the overall working of the technique.

### 2.3. DNA Isolation and PCR

For each experiment, genomic DNA was isolated from 30 colonies using the AccuPrep Genomic DNA Extraction kit (Bioneer Co., Daejeon, South Korea). The PCR reaction was carried out by using 20 μL of Bioneer premix (10 mM Tris-HCl, pH 9.0, 250 µM dNTP mix, 1.5 mM MgCl_2_, 40 mM KCl, and 1 U of *Taq* polymerase) [12].

### 2.4. Primer Synthesis and PCR

Primers for *Leu. mesenteroides* and *L. brevis* were designed using the primer BLAST (Basic Local Alignment Search Tool) from the National Center of Biotechnology Information database (NCBI) website (www.ncbi.nlm.nih.gov/BLAST). For *L. plantarum*, the primer sequences were used as described previously [25]. The primer synthesis was carried out by the Macrogen sequencing service (Seoul, South Korea). The amplification was performed in a BIORAD thermal cycler (Hercules, CA, USA). Gel imaging was carried out using the Bio-Rad Gel Doc XR+ gel documentation system. For fingerprint analysis, two DNA size markers, 100 bp (Bioneer, Daejeon, South Korea) and 1 kb (Takara, Japan), were used. The gel-purified (Wizard SV Gel and PCR Clean-Up system kits; Promega, USA) PCR products were sequenced by Bioneer Co. (Daejeon, South Korea).

### 2.5. 16 S rRNA Gene Sequencing and RAPD Analysis

The detailed procedure was performed as reported by reference [12].

### 2.6. Rep-PCR Analysis

To obtain the genomic fingerprints, the second typing method rep-PCR was performed using three primers: (GTG)_5_, enterobacterial repetitive intergenic consensus (ERIC), and repetitive extragenic palindromic (REP) for the analysis of 30 colonies for each experiment. The PCR procedure followed has been described previously [28]. Each reaction included 2 µL of DNA template, 1 µL (10 pmol/µL) of the reverse primer, 1 µL (10 pmol/µL) of the forward primer, and 16 µL of autoclaved distilled water to bring the total volume to 20 µL. The PCR products (5 µL) were then examined using 1.5% (*w*/*v*) agarose (Seakem, Lonza, Alpharetta, GA, USA) gel electrophoresis at 70 V for 5 h. The PCR amplification conditions for all the three rep-primers used in the present study are described in Table S1.

### 2.7. Comparative Sequence Analysis of the Housekeeping Genes

The third tracing method was based on the sequencing and analysis of the housekeeping genes. The genes, their products, and other relevant information for 11251, B151, and LB41 (LB41^K^ and LB41^P^) have been described in Tables S2–S4. Each PCR amplification experiment was carried out by using 1 µL of purified genomic DNA, 1 µL of the reverse primer, and 1 µL of the forward primer, and followed by the addition of autoclaved distilled water (17 µL) to make a final volume of 20 µL. The experiment was performed for the reference strains (11251, B151, and LB41) and the suspected colonies. The PCR amplification conditions are described in Table S1. The PCR products (5 µL) were electrophoresed using 1% (*w*/*v*) agarose gel at 70 V.

### 2.8. Tracing of the Intentionally Inoculated Strains

For each experiment, the 16S rRNA gene was amplified, sequenced, and BLAST-analyzed to retrieve the species-level information of the suspected colonies. After the species-level identification, the suspected colonies′ fingerprints were matched with the fingerprints of the reference strains (11251, B151, and LB41) using RAPD and Rep-PCR tools. Finally, the tracking was performed using comparative gene sequence analysis of the housekeeping genes through sequence trimming and alignments with the BioEdit [29] Sequence Alignment Editor (v. 7.2.5) and ClustalX v. 1.83 tools [30]. The colony without a single nucleotide polymorphisms (SNPs) was considered to be the inoculated reference strain.

The gene sequences from this study have been deposited at the GenBank, and their accession numbers are as follows: for 16S rRNA of *Leu. mesenteroides*, *L. brevis*, and *L. plantarum LB41*^K^: MF540920 to MF541009 and MF541070 to MF541098 for *L. plantarum* LB41^P^. Housekeeping gene sequences of *Leu. mesenteroides*: MG003189 to MG003195 (*atpA*), MG003211 to MG003217 (*pyrG*), MG003299 to MG003305 (*gyrB*), MG003321 to MG003327 (*groEL*), MG003255 to MG003261 (*pheS*), MG003277 to MG003283 (*rpoA*), MG003233 to MG003239 (*uvrC*); *L. brevis:* MF988109 to MF988117(*dnaK*), MF988120 to MF988128 (*groEL*), MF988131 to MF988139 (*gyrB*), MF988142 to MF988150 (*pheS*), MF988153 to MF988161 (*recA*), MF988164 to MF988172 (*rpoA*), MF988175 to MF988183 (*rpoB*); *L. plantarum* LB41^K^: MF988186 to MF988227; *L. plantarum* LB41^P^: MF988228 to MF988283.

## 3. Results

### 3.1. Analysis of 16S rRNA Sequences

For the Denmark drinking yogurt with unknown bacteria, out of the 30 colonies selected from the *Leu. mesenteroides*-inoculated culture, 22 colonies were identified as *S. thermophilus*, 7 (Y3, Y7, Y10, Y14–16, and Y27) colonies were identified as *Leu. mesenteroides*, and 1 colony (Y6) was identified as *L. plantarum* (Table 1). On the other hand, with Yakult yogurt (known bacteria), 21 out of 30 colonies from the *L. brevis*-inoculated yogurt were identified as *S. thermophilus*, whereas the remaining 9 colonies (YB2, YB4, YB9, YB15, YB18, YB21, YB23, YB25, and YB30) were identified as *L. brevis* (Table 1). From the *L. plantarum* LB41^K^-inoculated yogurt, 5 (YP5, YP6, YP9, YP15, and YP29) out of 30 colonies were identified as *L. plantarum*, whereas the remaining 25 colonies were identified as *S. thermophilus* (Table 1).

### 3.2. Analysis of RAPD-PCR Fingerprints

**Figure 2:**
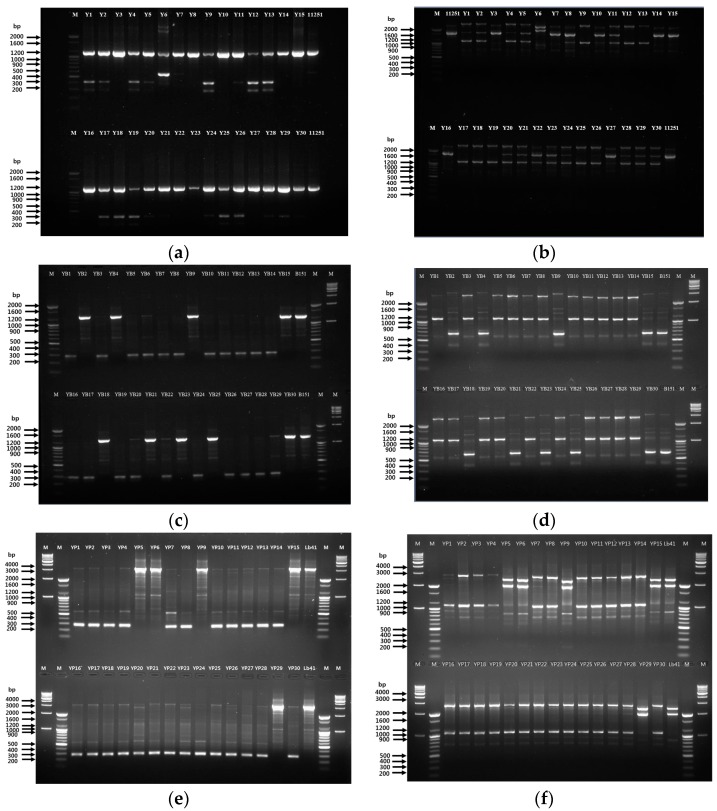
Tracing of the suspected colonies using RAPD-PCR analysis. Yogurt with strain 11251 (*Leu. mesenteroides*) using 239 primer (**a**); lane 1—marker 100 bp, lane 2–16 (colonies Y1–Y15), lane 17—11251 strain; lower half of the gel: lane 18—marker 100 bp, lane 19–33 (colonies Y16–Y30), lane 34—11251 strain; KAY3 primer (**b**); lane 1—marker 100 bp, lane 2—11251 strain, lane 3–17 (colonies Y1–15); lower half of the gel: lane 18—marker 100 bp, lane 19–33 (colonies Y16–30), lane 34—11251 strain. From yogurt with strain B151 (*L. brevis*) using 239 primer (**c**); KAY3 primer (**d**); lane 1—marker 100 bp, lane 2–16 (colonies YB1–15), lane 17—B151 strain, lane 18—marker 100 bp, lane 19—marker 1 kb; lower half of the gel: lane 20—marker 100 bp, lane 21–35 (colonies YB16–30), lane 36—B151 strain, lane 37—marker 100 bp, lane 38—marker 1 kb. From yogurt with strain LB41^K^ (*L. plantarum*) using 239 primer (**e**); KAY3 primer (**f**); lane 1—marker 1 kb, lane 2—marker 100 bp, lane 3–17 (colonies YP1–15), lane 18—LB41^K^ strain, lane 19—marker 100 bp, lane 20—marker 1 kb; lower half of the gel: lane 21—marker 1 kb, lane 22—marker 100 bp, lane 23–37 (colonies YP16–30), lane 38—LB41^K^ strain, lane 39—marker 100 bp, lane 40—marker 1 kb. Positive controls—11251, B151, and LB41^K^.

For each experiment, to trace the reference bacteria, the fingerprint profiles acquired with 239 and KAY3 RAPD primers were compared to fingerprints of the suspected colonies. In contrast to the 16S rRNA gene sequencing outcome, the primer 239 reactions displayed maximum colonies (24) that were likely to be similar to the reference strain, with a prominent band at 1100 bp (Figure 2a). However, the KAY3 primer matched the profile of seven suspected colonies, consisting of a major band at 1600 bp along with two light bands (Figure 2b). As expected, the identified colonies (using 16S rRNA gene sequencing) showed a *Leu. mesenteroides* 11251-specific banding pattern with both primers (Figure 2a,b).

However, few other colonies have also shown a similar banding profile to the control strain with the 239 primer. On the other hand, the tracing of *L. brevis* showed that nine colonies with a matching fingerprint profile to B151 strain had bright bands at 1200 and 660 bp with primers 239 (Figure 2c) and KAY3 (Figure 2d), respectively. Thus, these findings support the 16S rRNA BLAST results, suggesting a definite tracing of *L. brevis.* For the *L. plantarum* strain Lb41^K^, the RAPD assay revealed five colonies with a similar fingerprint and a bright band at 3000 bp using primer 239 (Figure 2e) and two major bands at 2000 and 2500 bp with the primer KAY3 (Figure 2f), which supports the results of the 16S rRNA sequencing analysis.

### 3.3. Analysis of Rep-PCR Fingerprints

**Figure 3:**
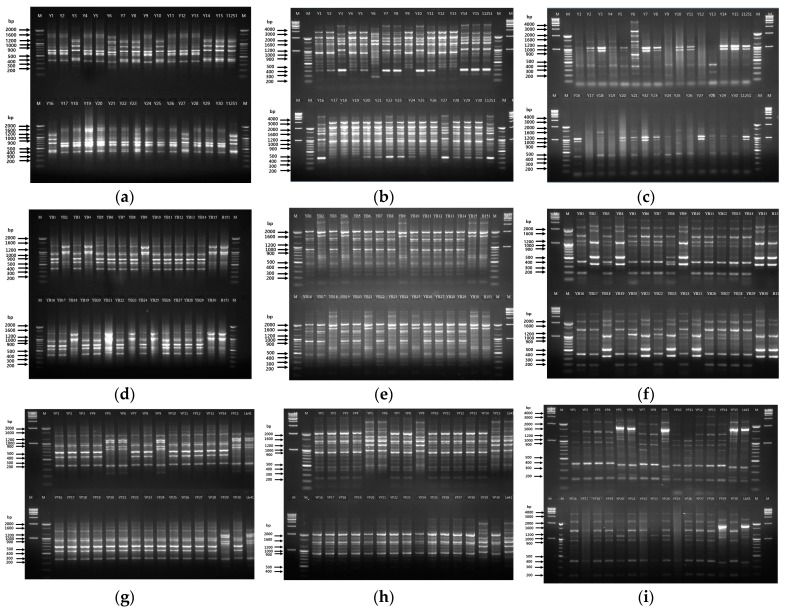
Tracing of the suspected colonies using rep-PCR analysis. Yogurt with strain 11251 (*Leu. mesenteroides*) using (GTG)_5_ primer (**a**); lane 1—marker 100 bp, lane 2–16 (colonies Y1–15), lane 17—11251 strain, lane 18—marker 100 bp; lower half of the gel: lane 19—marker 100 bp, lane 20–34 (colonies Y16–30), lane 35—11251 strain, lane 36—marker 100 bp. REP primer (**b**); and ERIC primer (**c**); lane 1—marker 1 kb, lane 2—marker 100 bp, lane 3–17 (colonies Y1–15), lane 18—11251 strain, lane 19—marker 100 bp, lane 20—marker 1 kb; lower half of the gel: lane 21—marker 1 kb, lane 22—marker 100 bp, lane 23–37 (colonies Y16–Y30), lane 38—11251 strain, lane 39—marker 100 bp, lane 40—marker 1 kb. Yogurt with strain B151 (*L. brevis*) using (GTG)_5_ primer (**d**); lane 1—marker 100 bp, lane 2–16 (colonies YB1–15), lane 17—B151 strain, lane 18—marker 100 bp; lower half of the gel: lane 19—marker 100 bp, lane 20–34 (colonies YB16–30), lane 35—B151 strain, lane 36—marker 100 bp. REP primer (**e**); lane 1—marker 100 bp, lane 2–16 (colonies YB1–15), lane 17—B151 strain, lane 18—marker 100 bp, lane 19—marker 1 kb; lower half of the gel: lane 20—marker 100 bp, lane 21–35 (colonies YB16–YB30), lane 36—B151 strain, lane 37—marker 100 bp, lane 38—marker 1 kb. ERIC primer (**f**); lane 1—marker 1 kb, lane 2—marker 100 bp, lane 3–17 (colonies YB1–15), lane 18—B151 strain, lane 19—marker 100 bp, lane 20—marker 1 kb; lower half of the gel: lane 21—marker 1 kb, lane 22—marker 100 bp, lane 23–37(colonies YB16–30), lane 38—B151 strain, lane 39—marker 100 bp, lane 40—marker 1 kb. Yogurt with strain LB41^K^ (*L. plantarum*) using (GTG)_5_ primer (**g**); REP primer (**h**); ERIC primer (**i**); lane 1—marker 1 kb, lane 2—marker 100 bp, lane 3–17 (colonies YP1–15), lane 18—strain LB41^K^, lane 19—marker 100 bp, lane 20—marker 1 kb; lower half of the gel: lane 21—marker 1 kb, lane 22—marker 100 bp, lane 23–37 (colonies YP16–30), lane 38—LB41^K^ strain, lane 39—marker 100 bp, lane 40—marker 1 kb. Positive controls—11251, B151, and LB41^K^.

Similar to RAPD, rep-PCR was used to trace the reference strains by comparing the banding profiles. In the yogurt inoculated with *Leu. mesenteroides*, the fingerprint pattern obtained for all three primers ((GTG)_5_, REP, and ERIC) were found to be identical to the pattern of the type strain for the seven identified colonies (Figure 3).

Among the three primers, the fingerprint profile with bands at 430, 700, 900, 1150, and 1200 bp positions was observed with (GTG)_5_ primer (Figure 3a). rep-PCR represented bands at 350, 1050, 1500, 1610, 2000, and 2500 bp in the reference as well as the suspected colonies (Figure 3b). With the ERIC primer, two main bands of 900 and 1050 bp were observed between the reference strain 11251 and seven suspected colonies (Figure 3c). These results confirmed that the seven colonies could be the inoculated strain 11251. On the other hand, parallel to RAPD analysis, nine colonies showed a matching banding profile to B151 with all rep-PCR primers (Figure 3).

With the (GTG)_5_ primer, three prominent bands at 1000, 1100, and 1500 bp were observed both in the reference and suspected bacterial colonies (Figure 3d). With REP-PCR, two bands at 1600 and 2000 bp positions were observed in B151 and nine colonies (Figure 3e). Likewise, the ERIC primer fingerprint pattern also showed a similar profile with bands at 350, 500, 1200, 2000, and 2200 bp (Figure 3f). The outcomes of the rep-PCR supported the RAPD analysis, thereby confirming that five colonies were similar to *L. plantarum* LB41^K^ (Figure 2). With the (GTG)_5_ primer, the bands can be seen at 350, 700, 800, and 1000–1200 bp positions both in the suspected colonies and LB41^K^ (Figure 3g). The REP primer displayed bands at 1000, 1200, 1500, 1700, 2100, and 4000 bp in LB41^K^ and the suspected colonies (Figure 3h). The ERIC primer produced bands at 380, 1700, and 2800 bp, which were similar in five colonies and LB41^K^ along with a few lighter bands (Figure 3i).

### 3.4. Comparative Housekeeping Gene Analysis

Finally, tracing of the inoculated strains was performed by comparing the partial sequence analysis of the seven housekeeping genes (Tables S2–S4). The colonies confirmed by the previously described molecular tools were used for the analysis. Therefore, the partial sequences of the housekeeping genes for *Leu. mesenteroides* (seven colonies), *L. brevis* (nine colonies), and of *L. plantarum* (five colonies) were compared and analyzed for the presence of SNPs with the reference strains. Alignment of the seven sequences with the respective partial gene sequences from the reference strain 11251 showed no SNPs in the seven identified colonies among *groEL*, *gyrB*, *atpA*, *pyrG*, *pheS*, *rpoA*, and *uvrC* housekeeping genes (Figure S1A–G). Similar results were obtained for the nine colonies, and all the nucleotide bases matched the consensus sequences of the B151 strain (Figure S2A–G); no SNPs were observed in any of the housekeeping (*gyrB*, *groEL*, *pheS*, *rpoB, dnaK, rpoA*, and *recA*) genes. Thus, we concluded that the suspected colonies, Y3, Y7, Y10, Y14–16, and Y27, were the initially inoculated *Leu. mesenteroides* strain 11251, and the nine suspected colonies, YB2, YB4, YB9, YB15, YB18, YB21, YB23, YB25, and YB30, were the *L. brevis* B151 reference strain. Likewise, the comparative gene sequence analysis of *ddl*, *gdh*, *gyrB*, *mutS*, *pgm*, *purK1*, and *tkt4* showed that each of the partial sequences matched with the sequence of LB41^K^ without any SNP in the housekeeping gene sequences (Figure S3A–G). This confirmed that the detected colonies, YP5, YP6, YP9, YP15, and YP29, were the LB41 ^K^ reference type that was initially inoculated in the yogurt.

### 3.5. Validation Using Tracing in a Probiotic Powder

For validation, tracing of *L. plantarum* (Lb41^P^) was evaluated in a probiotic powder consisting of a mixture of four bacterial species. As with the other inoculations, 30 colonies were randomly picked and labelled as PP1–30. The 16S rRNA gene sequencing results (Table 2) showed that seven (PP4, PP7, PP13, PP14, PP16, PP22, and PP25) of the 29 colonies were *L. plantarum*, whereas the rest of the colonies were identified as *S. thermophilus* and *L. acidophilus*. We could not obtain the 16S rRNA gene sequence for the PP8 colony.

**Figure 4:**
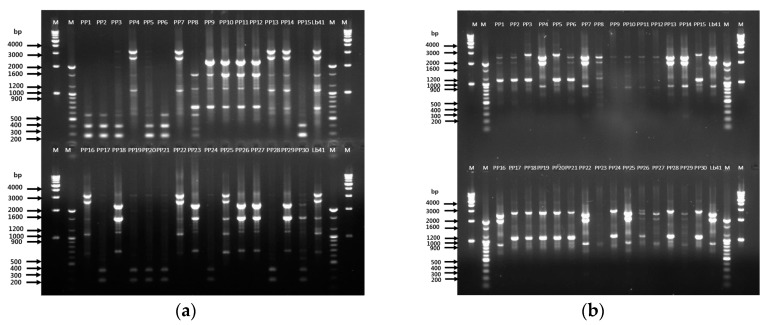
Tracing of the suspected colonies from probiotic powder using RAPD-PCR analysis. (**a**) Primer 239; (**b**) primer KAY3; lane 1—marker 1 kb, lane 2—marker 100 bp, lanes 3–17 (colonies PP1–PP15), lane 18—LB41^P^ strain, lane 19—marker 100 bp, lane 20—marker 1 kb; lower half of the gel: lane 21—marker 1 kb, lane 22—marker 100 bp, lanes 23–37 (colonies PP16–PP30), lane 38—LB41^P^ strain, lane 39—marker 100 bp, lane 40—marker 1 kb. Positive control—LB41^P^.

**Figure 5:**
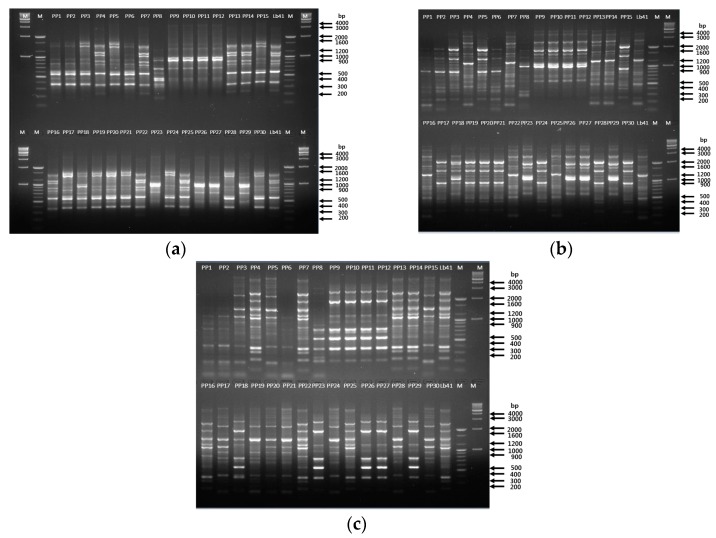
Tracing of the suspected colonies from probiotic powder using rep-PCR analysis. (**a**) Primer (GTG)_5_; lane 1—marker 1 kb, lane 2—marker 100 bp, lane 3–17 (colonies PP1–PP15), lane 18—LB41^P^ strain, lane 19—marker 100 bp, lane 20—marker 1 kb; lower half of the gel: lane 21—marker 1 kb, lane 22—marker 100 bp, lane 23–37 (colonies PP16–PP30), lane 38—LB41^P^ strain, lane 39—marker 100 bp, lane 40—marker 1 kb. REP primer (**b**); ERIC primer (**c**); lane 1–15 (colonies PP1–PP15), lane 16—LB41^P^ strain, lane 17—marker 100 bp, lane 18—marker 1 kb; lower half of the gel: lane 19–33 (colonies PP16–PP30), lane 34—LB41^P^ strain, lane 35—marker 100 bp, lane 36—marker 1 kb. Positive control—LB41^P^.

Furthermore, RAPD analysis displayed similar banding profiles with both primers. The primer 239 generated band profiles in the range of 700 to 3000 bp, whereas KAY3 generated bands in between 900 and 2500 bp (Figure 4a,b). Similar to the RAPD results, matched fingerprint profiles were obtained with rep-PCR primers (Figure 5). Figure 4a displays bands in the range of 350 to 2500 bp with the (GTG)_5_ primer, which matched the fingerprint profiles of seven colonies with Lb41^P^ (Figure 5a). Similarly, with primers REP and ERIC, the seven colonies displayed matching fingerprint profiles ranging from 200 to 5000 bp and 230 to 5000 bp, respectively (Figure 5b,c). To expand the analysis, we compared the partial gene sequences of seven housekeeping genes of *L. plantarum* (Table S4). After analysis, no SNPs were detected in any of the gene sequences; therefore, we concluded that the seven colonies were similar to the reference strain *L. plantarum* Lb41^P^ (Figure S4A–G).

## 4. Discussion

In summary, we focused our attention on the utilization of PCR-based approaches for the identification and tracking of selected LAB species. The results of this study have enhanced our knowledge on how various molecular typing methods could be utilized to track the desired bacterial strains in different food sources. The food industry has frequently been developing new products (a challenging task) to meet consumers’ demand under the stringent guidelines of various regulatory bodies [31]. Therefore, it necessitates the reliable and reproducible production of high-quality, stable, and safe products with a sufficient number of healthy bacteria, as described meticulously in a recent review by Fenster et al. [32]. The cutting-edge competition among industries requires the development of starters with novel properties for the generation of value-added products to meet customers′ needs. Furthermore, industries have to figure out many challenges such as designing, manufacturing, scaling up, maintaining safety standards and bacterial cultures, and commercialization of the products. If neglected, consumers may lose confidence in the products, hampering professional and economic credibility [33]. Therefore, the selection of wild LAB strains and their identification and characterization offer resources for product development, and also for the improvement of existing commercial processes. However, strains of commercial value may be misused by others for their commercial benefit. Therefore, molecular typing tools offer the dual advantage of analyzing the phylogeny of microbial isolates, and also can identify and trace commercially important strains.

The routine use of LAB species in industrial fermentations represents a billion-dollar industry [34]. Among dairy products, yogurt represents the most commonly consumed commodity along with cheese. In the present study, typing tools tracked seven colonies of *Leu. mesenteroides* in yogurt (unknown bacterial pool), which were identical to the reference strain, 11251. For the tracking of *L. brevis* B151 and *L. plantarum* LB41^K^ in yogurt (known pool—*S. thermophilus*, *B. longum*, *L. casei*, and *L. acidophilus*), nine and five colonies were identified, respectively. It is to be noted that *Leu. mesenteroides* is not generally used to produce commercial yogurt; however, some strains of *Leu. mesenteroides* have been used in the production of cheese [35]. Hence, as per the 16S rRNA gene sequencing results, it can be presumed that the colonies were the inoculated strain, *Leu. mesenteroides* 11251. However, a study by Chun et al. explained that 16S rRNA gene sequencing is not a suitable tool for inferring the phylogeny of *Leu. mesenteroides* strains [36]. Usually, also, 16S rRNA gene sequencing does not permit explicit separation of all bacterial strains at the species or subspecies level, which necessitates the use of other molecular tools [17,37]. In the RAPD analysis, most of the colonies displayed identical banding profiles to the 11251 strain with primer 239, which could be due to the similar binding sites for the primer in the genomes isolated from the 30 colonies. In Mechnikop yogurt, three (*B. longum*, *L. casei*, and *L. acidophilus*) of the four species were not detected, which reflects that these species require supplemented growth media and different culture conditions to grow. *Bifidobacterium* spp. generally prefer to grow MRS/MRS-nalidixic acid, paromomycin, neomycin sulphate, and lithium chloride (NPNL) at 37 °C for 72 h under anaerobic conditions. *L. acidophilus* shows selective growth on MRS agar supplemented with maltose (MRSM) or 5-bromo-4-chloro-3-indolyl-β-d-glucopyranoside (X-Glu) followed by anaerobic incubation. On the other hand, *L. casei* can grow on selective medium containing ribose (1% w/v) LC agar, anaerobic incubation at 27 °C. In addition, many other media have also been reported for the enumeration of these aforementioned bacterial species [38]. The remaining colonies, other than those suspected, were identified as *S. thermophilus*. Furthermore, the bacterial pool in the yogurt (Korea Yakult) inoculated with *L. brevis* and *L. plantarum* LB41^K^ strains were already known (marked on the label), and it was certain that the yogurt was not made by utilizing any of the *L. brevis* and *L. plantarum* strains. Thus, the identified colonies should be the inoculated strains, B151 and *L. plantarum* LB41^K^.

PCR-based fingerprinting tools such as RAPD [12] and rep-PCR [39] had parallel discriminatory powers and appeared apposite for the distinction of bacteria. However, the pitfalls include reproducibility and comparability between different research laboratories [40]. Therefore, the necessity of identification and higher discrimination of LAB has led to the use of housekeeping gene sequence analysis [41,42]. Research by Shevtsov et al. showed that the identification of the *Lactobacillus* genus using housekeeping gene sequences is superior and more sensitive than the 16S rRNA gene sequencing [43]. Previously, our group characterized different strains of *Leu. mesenteroides* and *L. brevis* from Korea using the multilocus sequence typing (MLST) molecular tool [44]. The same set of gene sequences were utilized for the comparative gene sequence analysis to identify the deliberately inoculated *Leu. mesenteroides* 11251 and *L. brevis* B151 in yogurt [45,46]. For the *L. plantarum* strains, the housekeeping genes were used from an earlier described MLST study [25].

In industrial fermentations, *L. plantarum* is the most commonly used bacterial species as a microbial starter or probiotic bacteria [47]. Therefore, to validate the approach, the tracing of a *L. plantarum* strain from a commercial probiotic powder was investigated by identifying the LB41^P^ strain. All four molecular tools effectively identified seven colonies as the reference strain. These results confirm the utility of the developed technology for the tracking of desired bacterial strains.

Capillary sequencing (also known as Sanger sequencing) is a fast and cost-effective technology that is suitable for a low number of targets such as cloned DNA fragments or PCR products. It suffers challenges such as low sensitivity, high cost per sample for large number of targets, and challenging to scale. On the other hand, using next-generation technologies (NGS), one can reach a better conclusion about the identification of the suspected strain. The important criteria of this technology include read length, quality of the sequence, and cost. Ideally, the sequence data should be with long read length and low error rates. However, there is no report of such a technology. This technology is favored where novel or unique variants of the bacteria are required. Moreover, NGS is a labor-intensive, costly affair that requires technical expertise with heavy and costly equipment. Moreover, approximately 4000 USD is required to sequence the complete genome of probiotic bacteria [33]. Moreover, not all probiotic industrial strains have been sequenced. Therefore, NGS use is not feasible for every industry (especially small-scale industry) or laboratory. In contrast, the tools used in this study are comparatively easy, cost-effective, and can be used in the identification of dubious strains.

Flow cytometry (FC) is a quick and automated method for enumeration, detection, and microbial profiling. However, the identity of microorganisms depends upon the use of different probes and fluorescent dyes. Also, the cost of this sophisticated and expensive instrument and technical operator is much higher compared to the tools used in the present technology. On the other hand, analysis time for FC is faster, around 15–20 min compared to 2–5 days for culturing of bacteria [48]. Recently, FC has been utilized for the viability assessment and quantification of microorganisms in multi-strain probiotic products [49]. Mass spectrometry is a fast and less labor-intensive technique for bacterial identification. The approximate cost was reported to be 0.5 to 1.00 USD per sample [50]. However, pretreatment of chemicals, temperature, and media composition may affect the quality of spectra. In addition, the technique is suitable for pure cultures only, and provides information at the genus level, not at the species or subspecies level [51]. Nonetheless, these methods are still research-based and costs vary depending upon the number of samples for analysis.

In our research, all the molecular methods were proven to be practical tools in the tracing of intentionally inoculated strains. In addition, the yogurt utilized was not made from any of the LAB strains selected for the present study. Nonetheless, as discussed earlier, all the molecular tools have some disadvantages; therefore, the identification of the desired strain cannot depend on a particular method and should be followed by a combined approach. Notably, the selection of the typing method depends on the objective of the research, the availability of skilled personnel, and, most importantly, the resources in the laboratory. It is a fact that the comparison of housekeeping gene sequences is a measured approach. However, new alternatives such as whole-genome sequencing (WGS) and whole-genome MLST (wgMLST) are becoming popular (subject to affordability) for the description and identification of a bacterial species. Further investigations should be focused on the utilization of these technologies.

## 5. Strengths and Limitations of the Study

The strength of the present study includes the use of practically viable molecular tools such as RAPD, rep-PCR, and comparative gene sequence analysis for the identification of three LAB species. Moreover, the technology was validated by identifying target bacterial species from a commercial product. In addition, the presented cost-effective technology can be easily performed in a basic molecular biology lab. It can be used for the identification of bacteria in most food products. The limitations of the study include the time-consuming culture dependency and the selection of food products without the presence of target reference strains.

## 6. Conclusions

Molecular tools for identifying microorganisms have been emerging in recent decades. Nonetheless, many limitations such as high running costs, skilled workforce, and expensive equipment need to be overcome for many of these newly developed technologies. Herein, our approach describes a technology with low cost and simple instrumentation for the identification of LAB. Our analysis of the 30 random selections and corresponding reference strains showed the practical feasibility of the approach for identifying suspected LAB strains. These PCR-based molecular tools showed efficacy for the identification of suspected *Leu. mesenteroides*, *L. brevis*, and *L. plantarum* (LB41^P^) in commercial yogurt, and substantiated viability by identifying *L. plantarum* (LB41^K^) in a commercial probiotic powder. Overall, the union of these low-cost molecular tools would help users for the identification of suspected LAB or other probiotics strains.

## Figures and Tables

**Table 1:** Results of the 16S rRNA gene sequencing for *Leu. mesenteroides* (11251), *L. brevis* (B151), and *L. plantarum* (LB41^K^) strains inoculated in yogurt.

Serial No.	Colony ID	BLAST * Result	Colony ID	BLAST Result	Colony ID	BLAST Result
1.	Y1	*S. thermophilus*	YB1	*S. thermophilus*	YP1	*S. thermophilus*
2.	Y2	*S. thermophilus*	YB2	*L. brevis*	YP2	*S. thermophilus*
3.	Y3	*Leu. mesenteroides*	YB3	*S. thermophilus*	YP3	*S. thermophilus*
4.	Y4	*S. thermophilus*	YB4	*L. brevis*	YP4	*S. thermophilus*
5.	Y5	*S. thermophilus*	YB5	*S. thermophilus*	YP5	*L. plantarum*
6.	Y6	*L. plantarum*	YB6	*S. thermophilus*	YP6	*L. plantarum*
7.	Y7	*Leu. mesenteroides*	YB7	*S. thermophilus*	YP7	*S. thermophilus*
8.	Y8	*S. thermophilus*	YB8	*S. thermophilus*	YP8	*S. thermophilus*
9.	Y9	*S. thermophilus*	YB9	*L. brevis*	YP9	*L. plantarum*
10.	Y10	*Leu. mesenteroides*	YB10	*S. thermophilus*	YP10	*S. thermophilus*
11.	Y11	*S. thermophilus*	YB11	*S. thermophilus*	YP11	*S. thermophilus*
12.	Y12	*S. thermophilus*	YB12	*S. thermophilus*	YP12	*S. thermophilus*
13.	Y13	*S. thermophilus*	YB13	*S. thermophilus*	YP13	*S. thermophilus*
14.	Y14	*Leu. mesenteroides*	YB14	*S. thermophilus*	YP14	*S. thermophilus*
15.	Y15	*Leu. mesenteroides*	YB15	*L. brevis*	YP15	*L. plantarum*
16.	Y16	*Leu. mesenteroides*	YB16	*S. thermophilus*	YP16	*S. thermophilus*
17.	Y17	*S. thermophilus*	YB17	*S. thermophilus*	YP17	*S. thermophilus*
18.	Y18	*S. thermophilus*	YB18	*L. brevis*	YP18	*S. thermophilus*
19.	Y19	*S. thermophilus*	YB19	*S. thermophilus*	YP19	*S. thermophilus*
20.	Y20	*S. thermophilus*	YB20	*S. thermophilus*	YP20	*S. thermophilus*
21.	Y21	*S. thermophilus*	YB21	*L. brevis*	YP21	*S. thermophilus*
22.	Y22	*S. thermophilus*	YB22	*S. thermophilus*	YP22	*S. thermophilus*
23.	Y23	*S. thermophilus*	YB23	*L. brevis*	YP23	*S. thermophilus*
24.	Y24	*S. thermophilus*	YB24	*S. thermophilus*	YP24	*S. thermophilus*
25.	Y25	*S. thermophilus*	YB25	*L. brevis*	YP25	*S. thermophilus*
26.	Y26	*S. thermophilus*	YB26	*S. thermophilus*	YP26	*S. thermophilus*
27.	Y27	*Leu. mesenteroides*	YB27	*S. thermophilus*	YP27	*S. thermophilus*
28.	Y28	*S. thermophilus*	YB28	*S. thermophilus*	YP28	*S. thermophilus*
29.	Y29	*S. thermophilus*	YB29	*S. thermophilus*	YP29	*L. plantarum*
30.	Y30	*S. thermophilus*	YB30	*L. brevis*	YP30	*S. thermophilus*

* BLAST: Basic Local Alignment Search Tool; *Leuconostoc* (*Leu.*) *mesenteroides*; *Lactobacillus* (*L.*) *brevis*; *Streptococcus* (*S.*) *thermophilus*; *Lactobacillus* (*L.*) *plantarum*.

**Table 2:** Results of the 16S rRNA gene sequencing for *L. plantarum* (LB41^p^) identified from probiotic powder.

Serial No.	Colony ID	BLAST Result	Serial No.	Colony ID	BLAST Result
1.	PP1	*S. thermophilus*	16.	PP16	*L. plantarum*
2.	PP2	*S. thermophilus*	17.	PP17	*S. thermophiles*
3.	PP3	*S. thermophilus*	18.	PP18	*L. acidophilus*
4.	PP4	*L. plantarum*	19.	PP19	*S. thermophilus*
5.	PP5	*S. thermophilus*	20.	PP20	*S. thermophilus*
6.	PP6	*S. thermophilus*	21.	PP21	*S. thermophilus*
7.	PP7	*L. plantarum*	22.	PP22	*L. plantarum*
8.	PP8 *	*-------------*	23.	PP23	*L. acidophilus*
9.	PP9	*L. acidophilus*	24.	PP24	*S. thermophilus*
10.	PP10	*L. acidophilus*	25.	PP25	*L. plantarum*
11.	PP11	*L. acidophilus*	26.	PP26	*L. acidophilus*
12.	PP12	*L. acidophilus*	27.	PP27	*L. acidophilus*
13.	PP13	*L. plantarum*	28.	PP28	*S. thermophilus*
14.	PP14	*L. plantarum*	29.	PP29	*L. acidophilus*
15.	PP15	*S. thermophilus*	30.	PP30	*S. thermophilus*

* Sequence data was not available; BLAST: Basic Local Alignment Search Tool; *Lactobacillus (L.) plantarum*; *Streptococcus* (*S.*) *thermophilus*; *Lactobacillus* (*L.*) *acidophilus*.

## Supplementary Materials

The following are available online at https://www.mdpi.com/2076-2607/8/1/5/s1: Figure S1: Comparative gene sequence analysis—*groEL* (A), *gyrB* (B), *atpA* (C), *pyrG* (D), *pheS* (E), *rpoA* (F), and *uvrC* (G) of seven colonies isolated from yogurt inoculated with reference strain 11251 (*Leu. mesenteroides*). Figure S2: Comparative gene sequence analysis—*gyrB* (A), *groEL* (B), *pheS* (C), *rpoB* (D)*, dnaK* (E), *rpoA* (F)**, and *recA* (G) of nine colonies isolated from yoghurt inoculated with reference strain B151 (*L. brevis*). Figure S3: Comparative gene sequence analysis—*ddl* (A), *gdh* (B), *gyrB* (C), *mutS* (D), *pgm* (E), *purK1* (F), and *tkt4* (G) of five colonies isolated from yogurt inoculated with the reference strain LB41^K^ (*L. plantarum*). Figure S4: Comparative gene sequence analysis—*ddl* (A), *gdh* (B), *gyrB* (C), *mutS* (D), *pgm* (E), *purK1* (F), and *tkt4* (G) of seven colonies isolated from probiotic powder with the reference strain LB41^P^ (*L. plantarum*). Table S1: PCR conditions used for rep-PCR and for the amplification of housekeeping genes of the target colonies. Table S2: Information of housekeeping loci and primers used for *Leu. mesenteroides* 11251 strain. Table S3: Information of housekeeping loci and primers used for *L. brevis* B151 strain. Table S4: Information of housekeeping loci and primers used for *L. plantarum* LB41 strains.
