# Supplementary material for: Tracking of Intentionally Inoculated Lactic Acid Bacteria Strains in Yogurt and Probiotic Powder
Source: Microorganisms. 2019 Dec 18;8(1):5. doi: 10.3390/microorganisms8010005 (PMC7022703; doi:10.3390/microorganisms8010005)

Tracking of intentionally inoculated lactic acid bacteria strains in yogurt and probiotic powder

Anshul Sharma^1,2,3^, Jasmine Kaur^1^, Sulhee Lee^1^, and Young-Seo Park^1^**^*^**

1. Department of Food Science and Biotechnology, Gachon University, Gyeonggi-do 13120, Republic of Korea

2. Department of Food and Nutrition, Gachon University, Gyeonggi-do 13120, Republic of Korea

3. Faculty of Applied Sciences and Biotechnology, Shoolini University of Biotechnology and Management Sciences, Bajhol, Solan, Himachal Pradesh 173229, India

**^*^**Corresponding author

E-mail address: ypark@gachon.ac.kr (Prof. Y.-S. Park)

Mobile: +821088675378

**Supplementary Figures**

**Figure S1.** Comparative gene sequence analysis- *groEL* (A), *gyrB* (B), *atpA* (C), *pyrG* (D), *pheS* (E), *rpoA* (F), and *uvrC* (G) of 7 colonies isolated from yogurt inoculated with reference strain 11251 (*Leu. mesenteroides*).


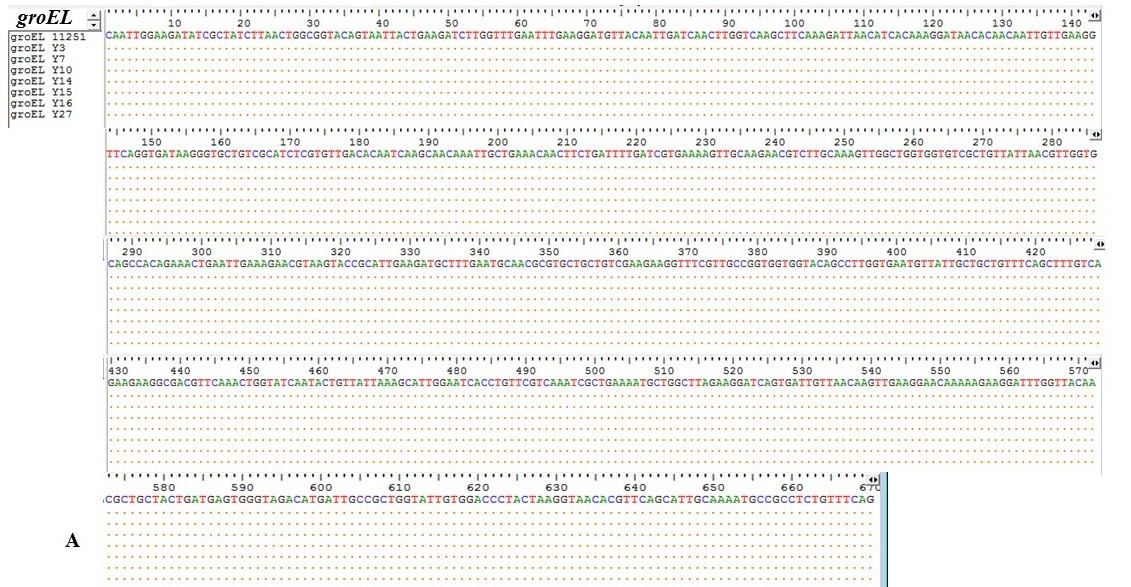


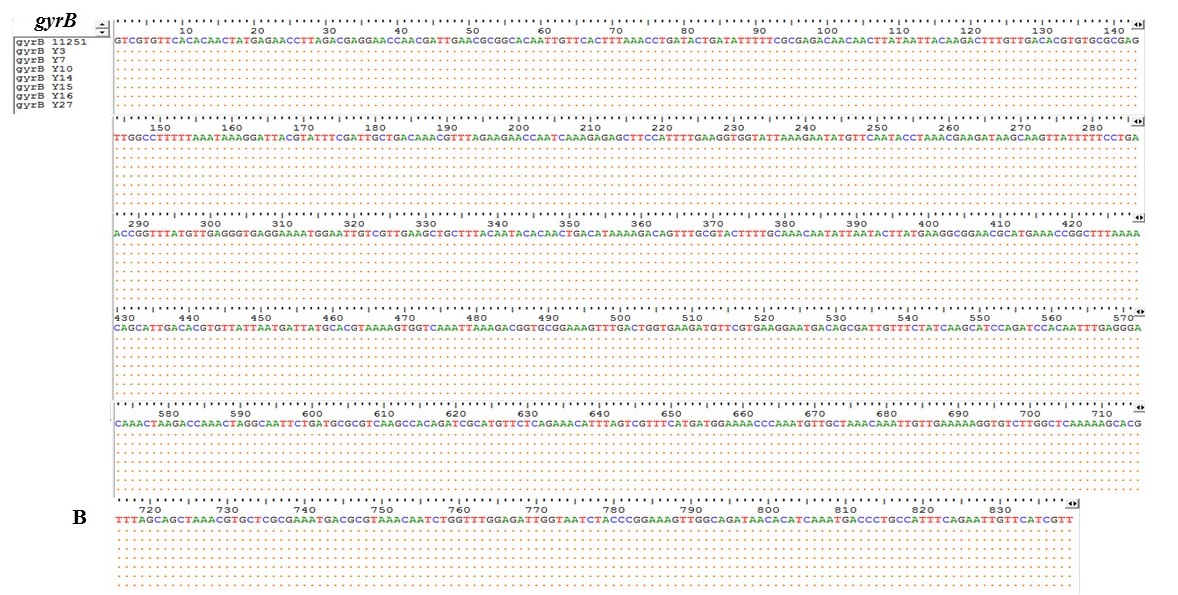


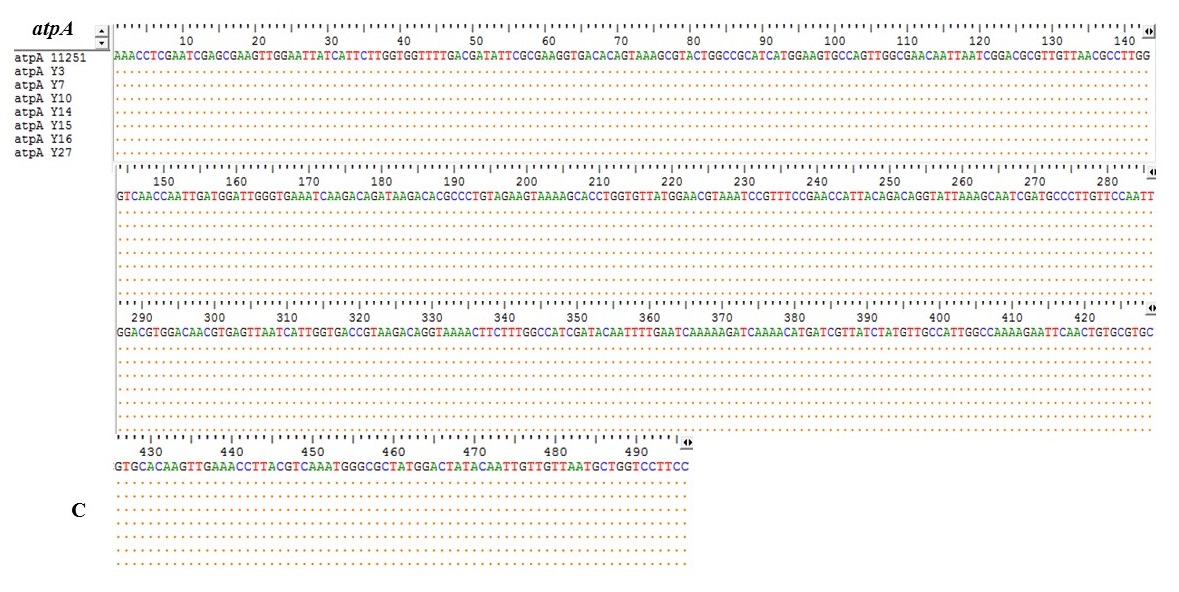


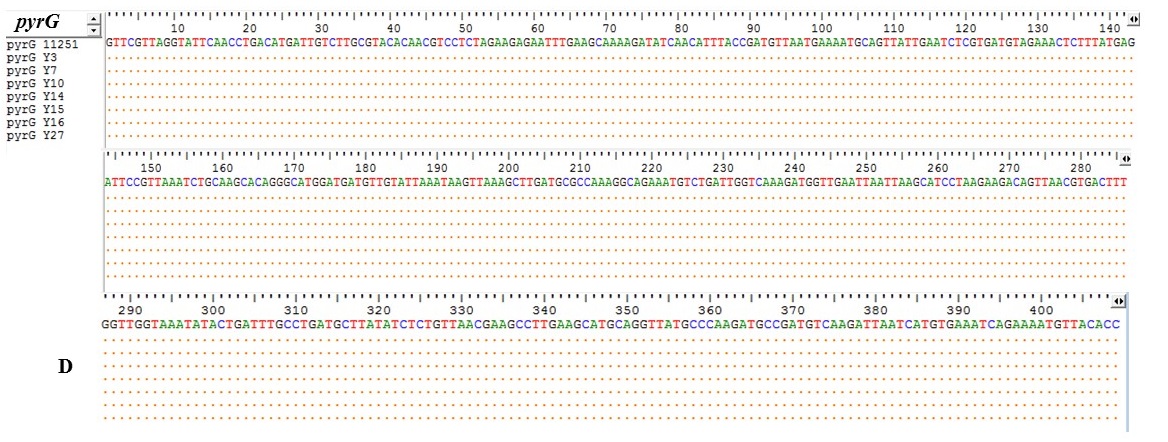


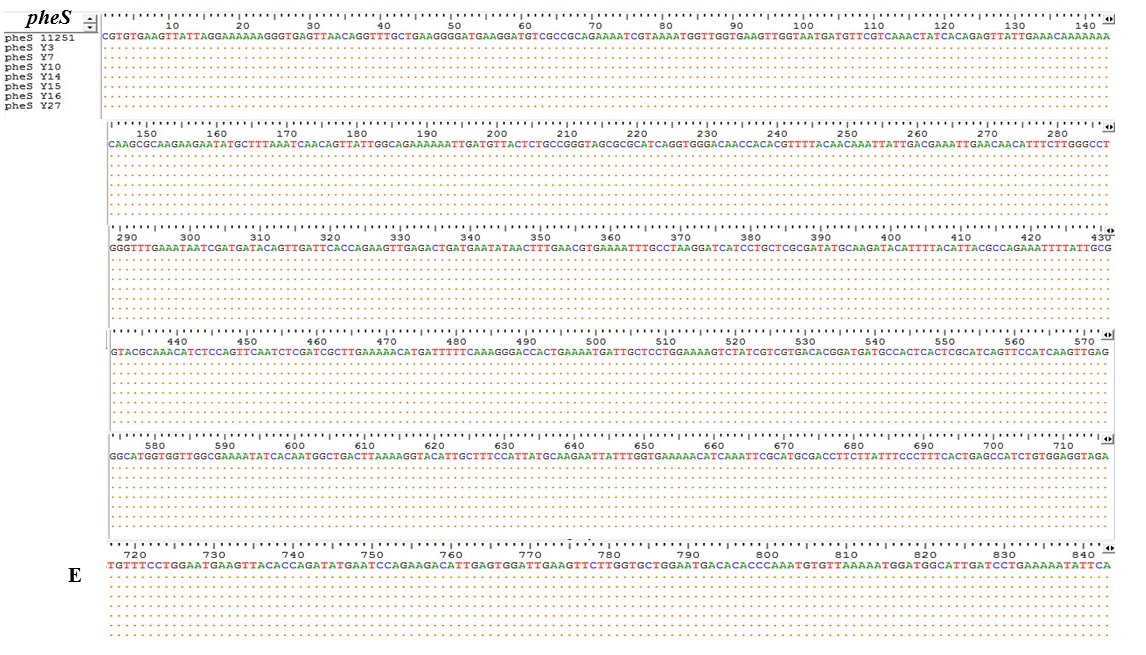


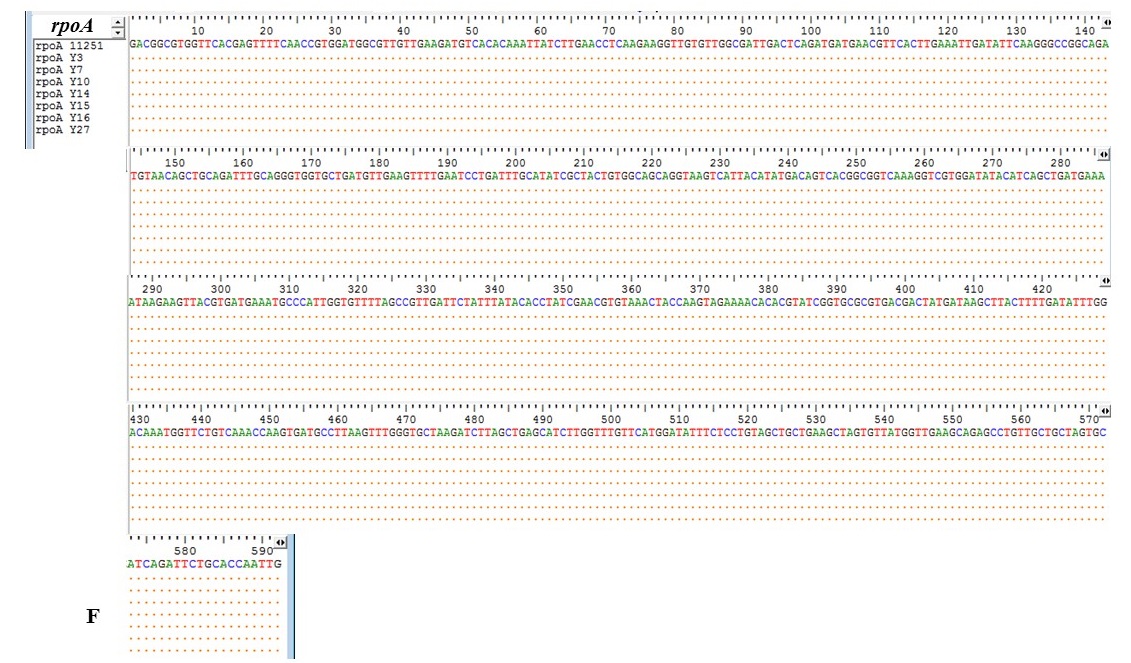


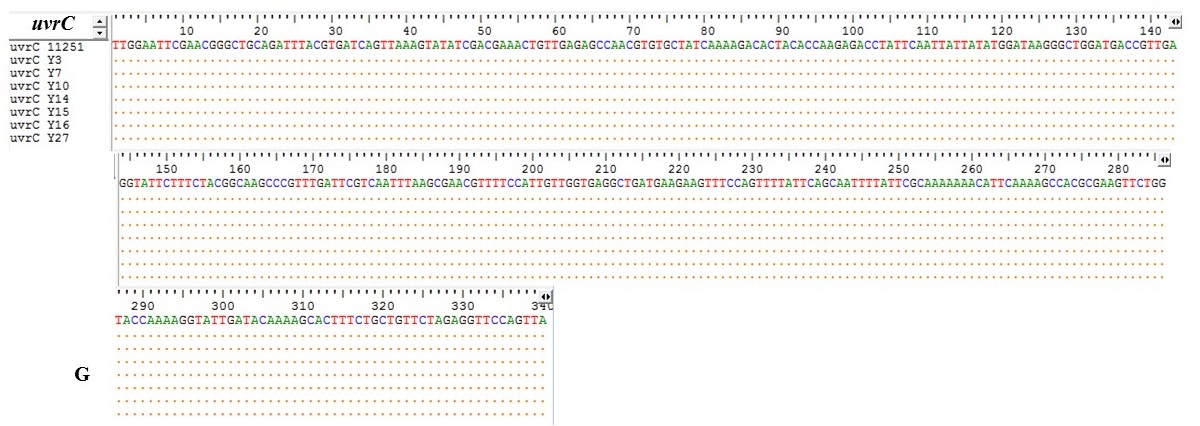


**Figure. S2** Comparative gene sequence analysis- *gyrB* (A), *groEL* (B), *pheS* (C), *rpoB* (D)*, dnaK* (E), *rpoA* (F)*,* and *recA* (G) of 9 colonies isolated from yogurt inoculated with reference strain B151(*L. brevis*).


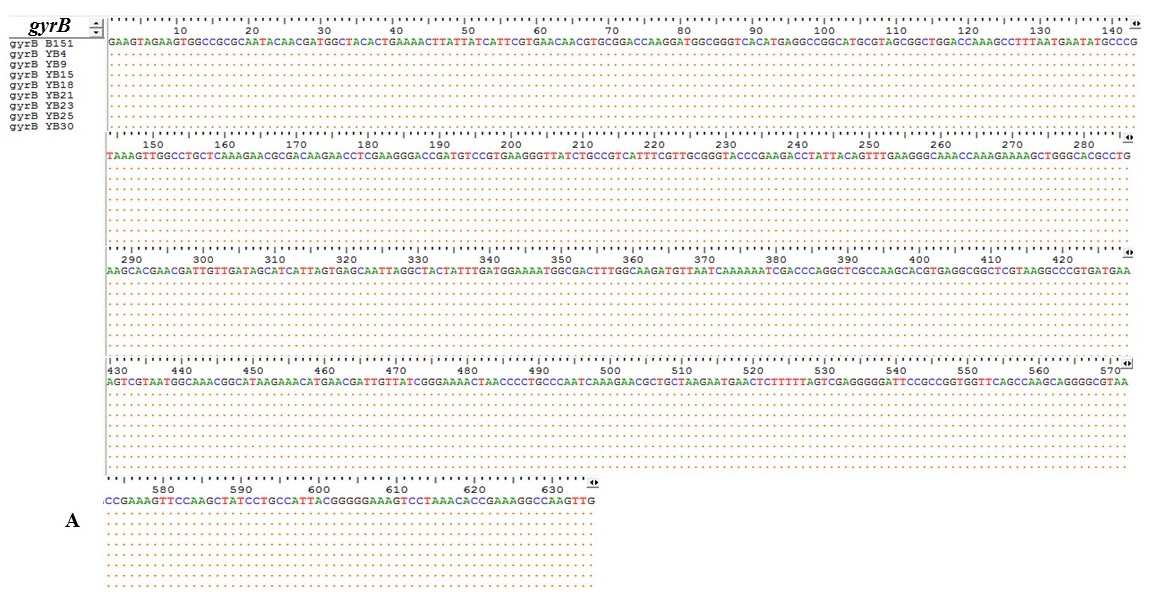


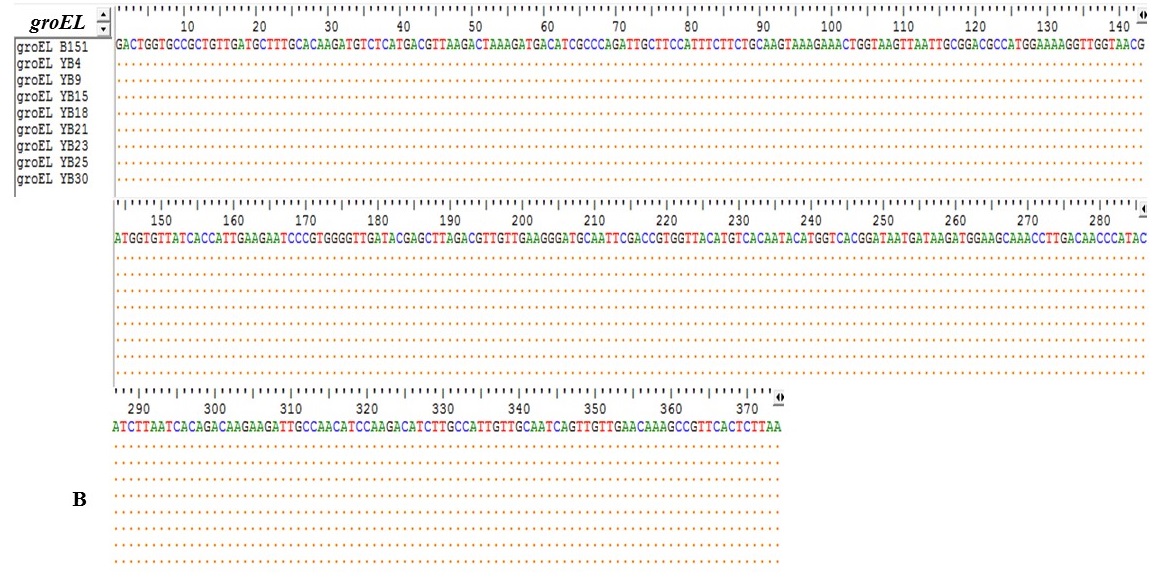


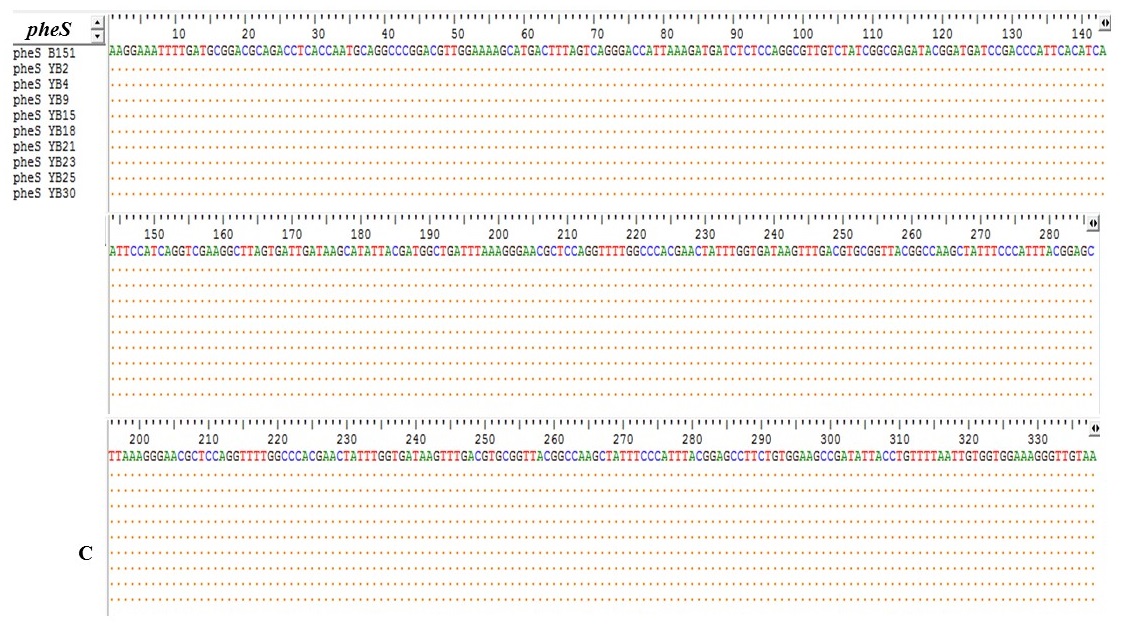


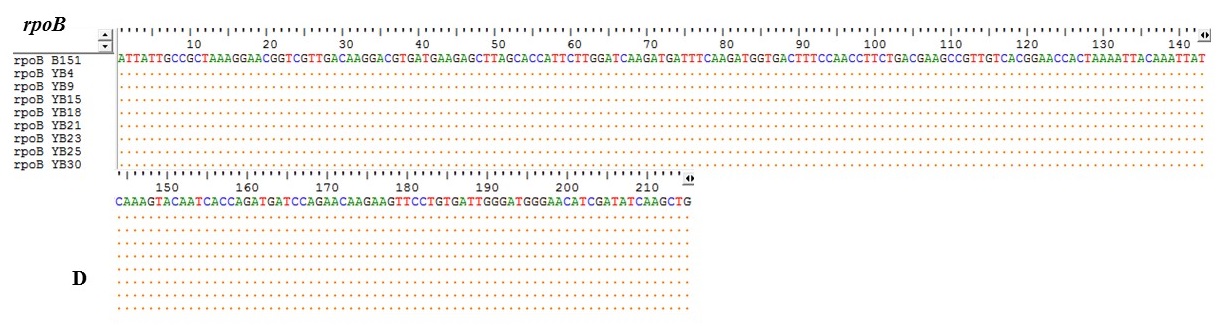


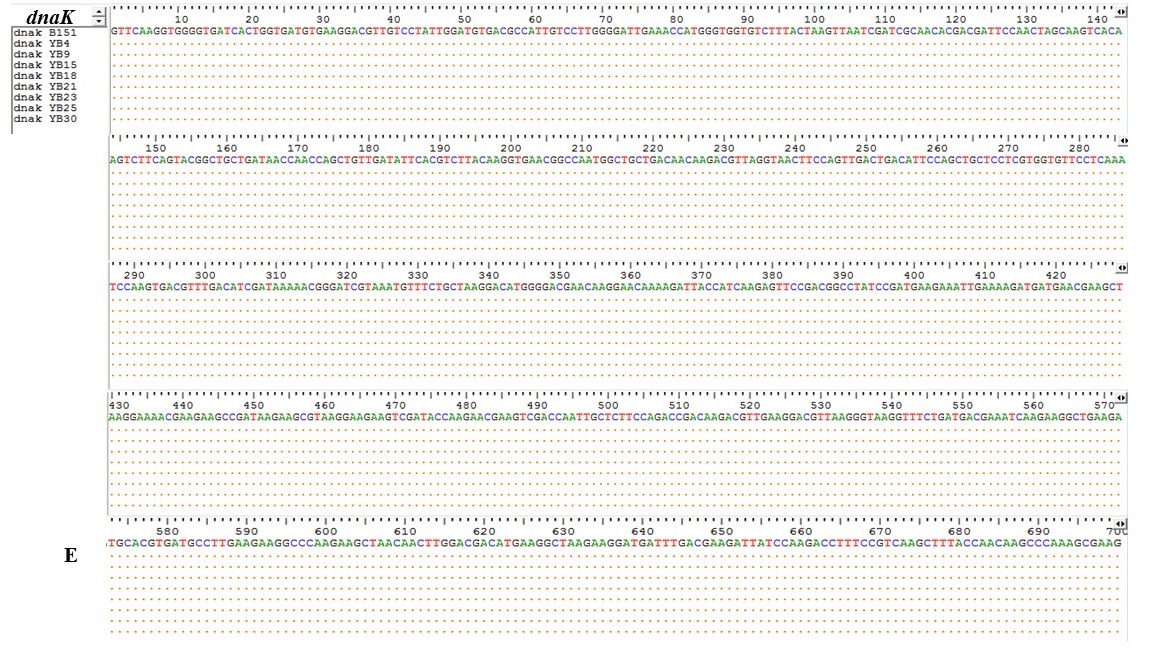


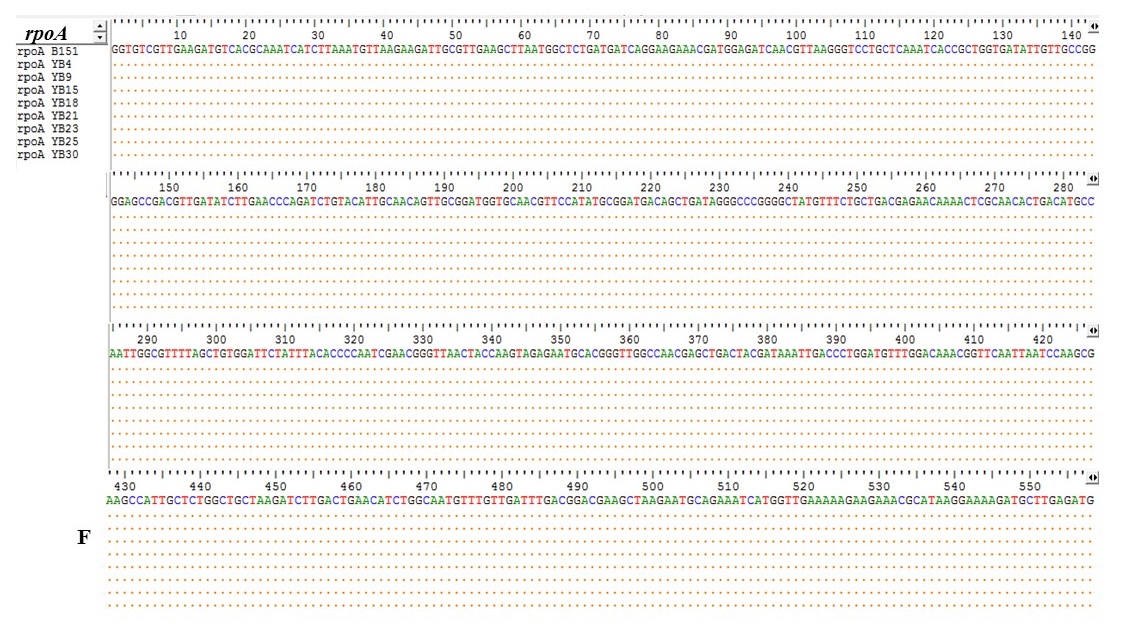


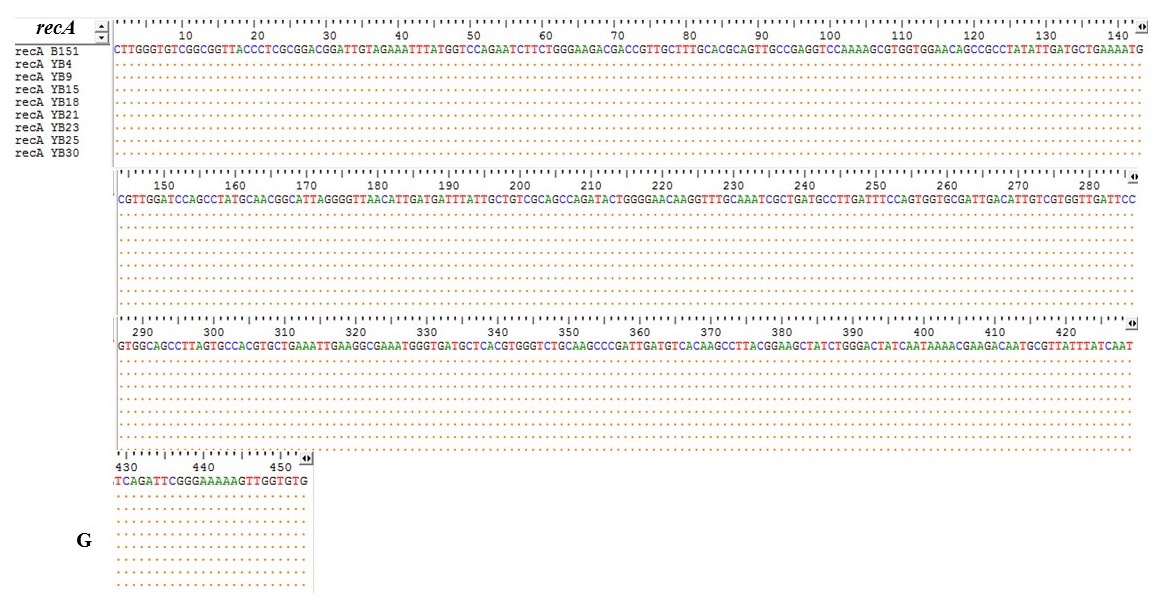


**Figure. S3** Comparative gene sequence analysis- *ddl* (A), *gdh* (B), *gyrB* (C), *mutS* (D), *pgm* (E), *purK1* (F) and *tkt4* (G) of 5 colonies isolated from yogurt inoculated with reference strain LB41^K^ (*L. plantarum*).


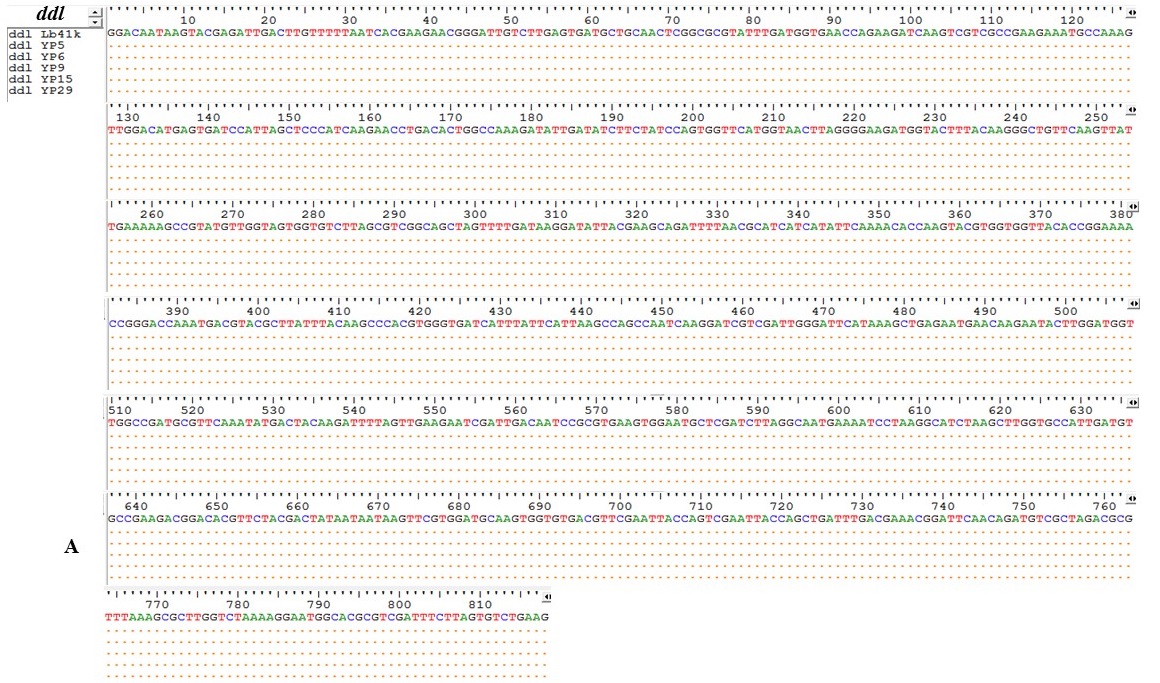


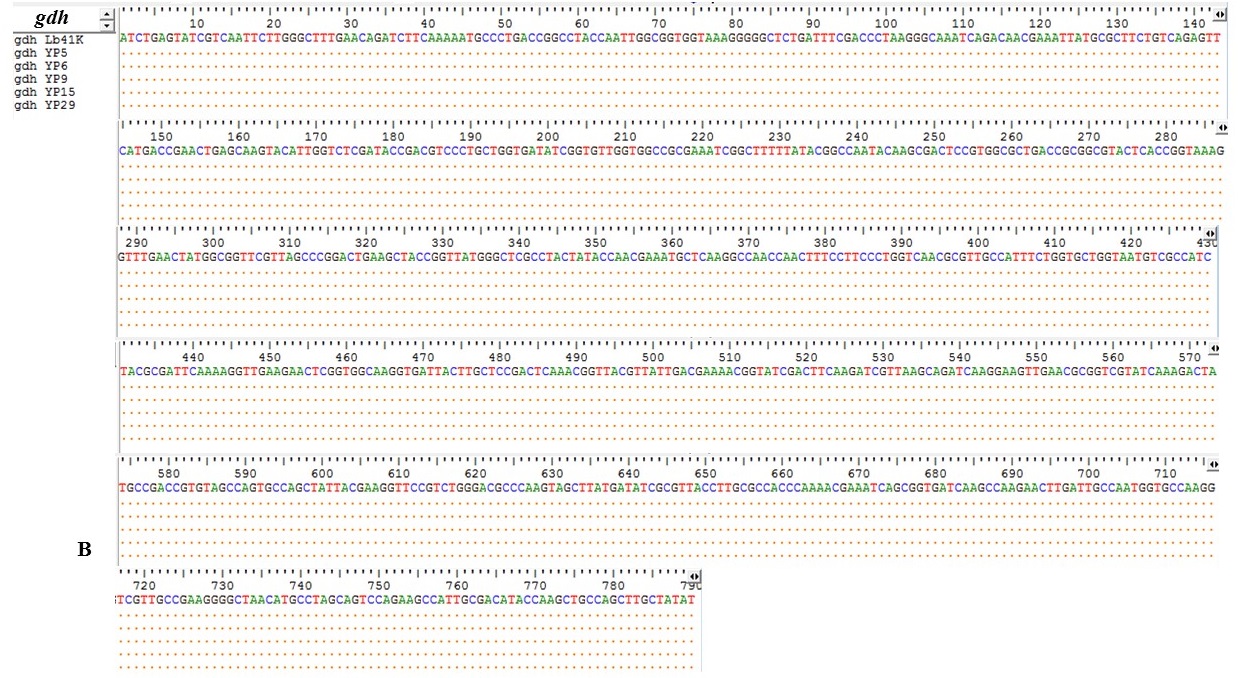


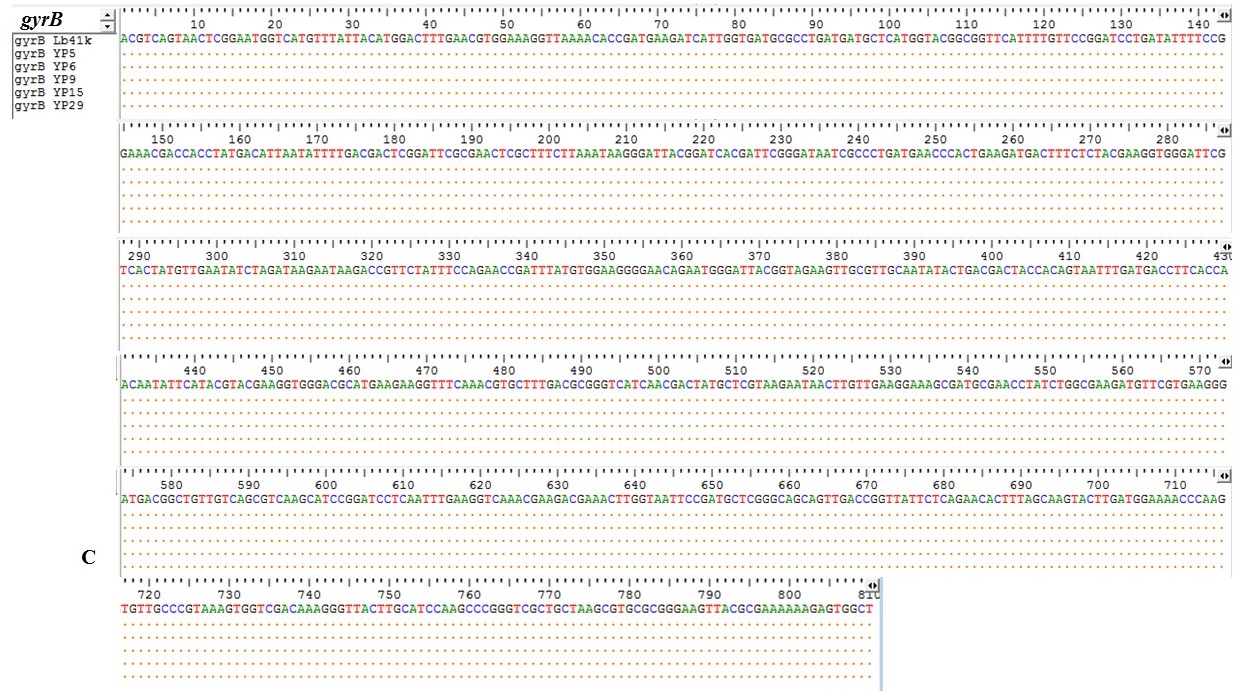


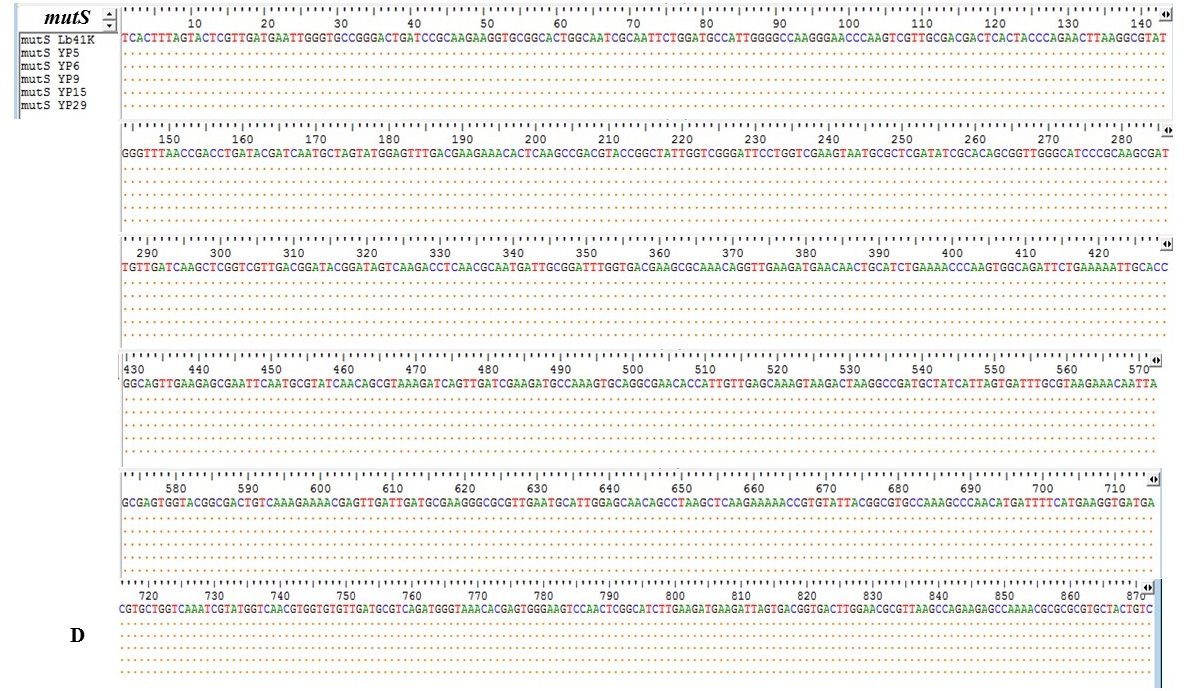


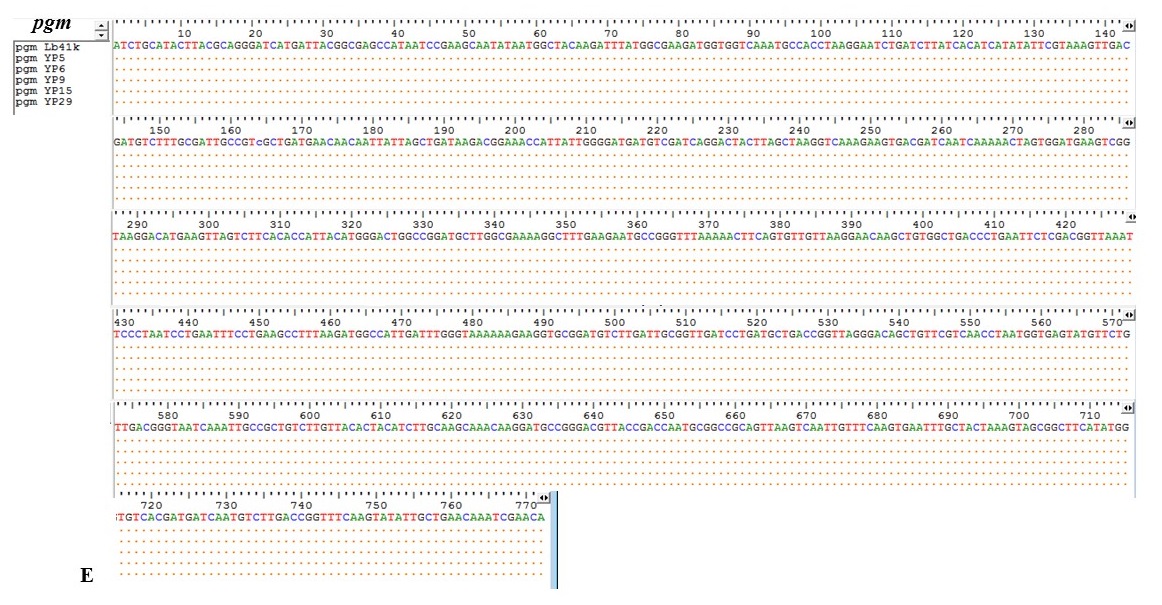


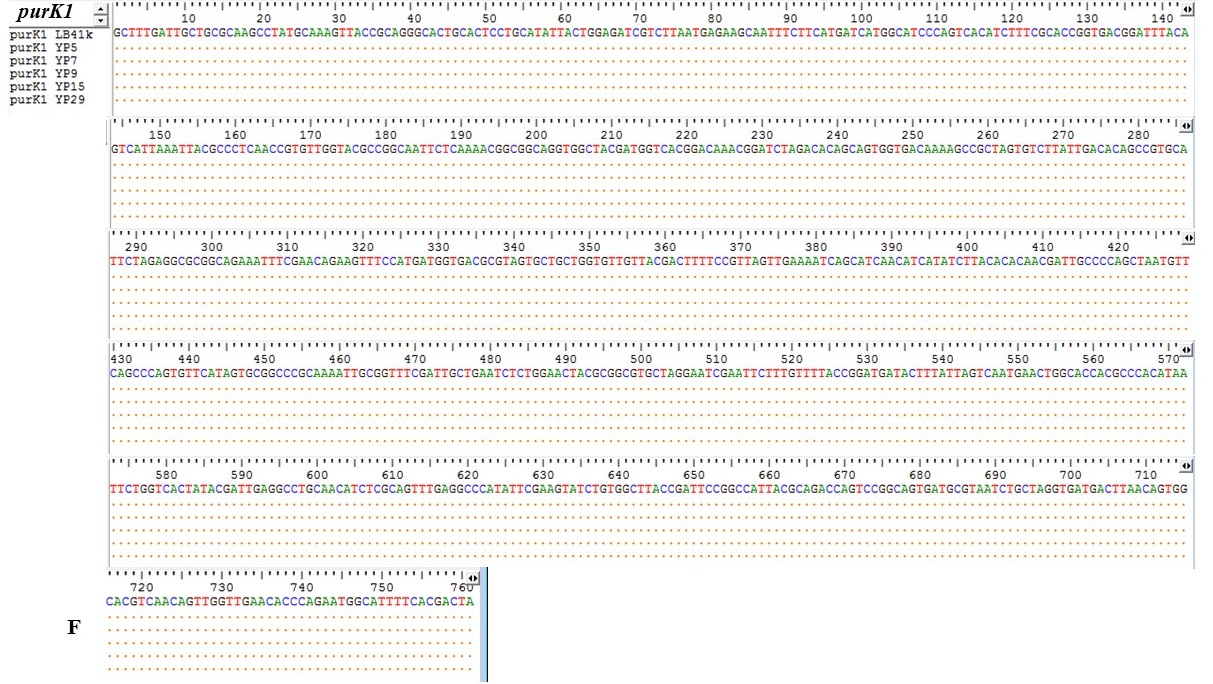


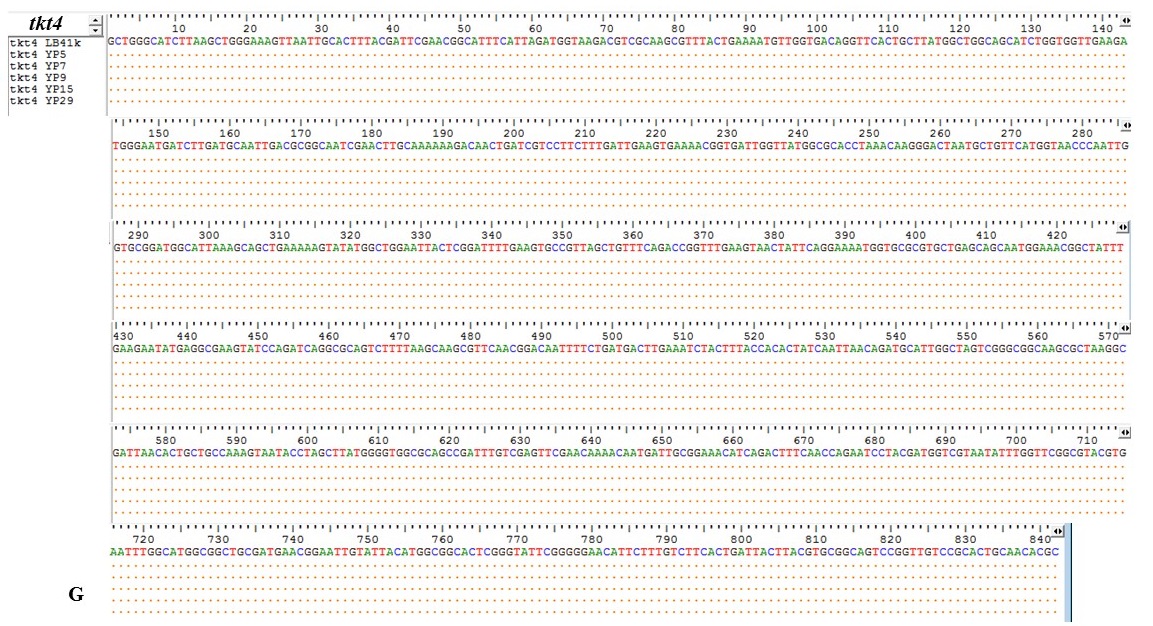


**Figure. S4** Comparative gene sequence analysis- *ddl* (A), *gdh* (B), *gyrB* (C), *mutS* (D), *pgm* (E), *purK1* (F) and *tkt4* (G) of 7 colonies isolated from probiotic powder with reference strain LB41^P^ (*L. plantarum*).


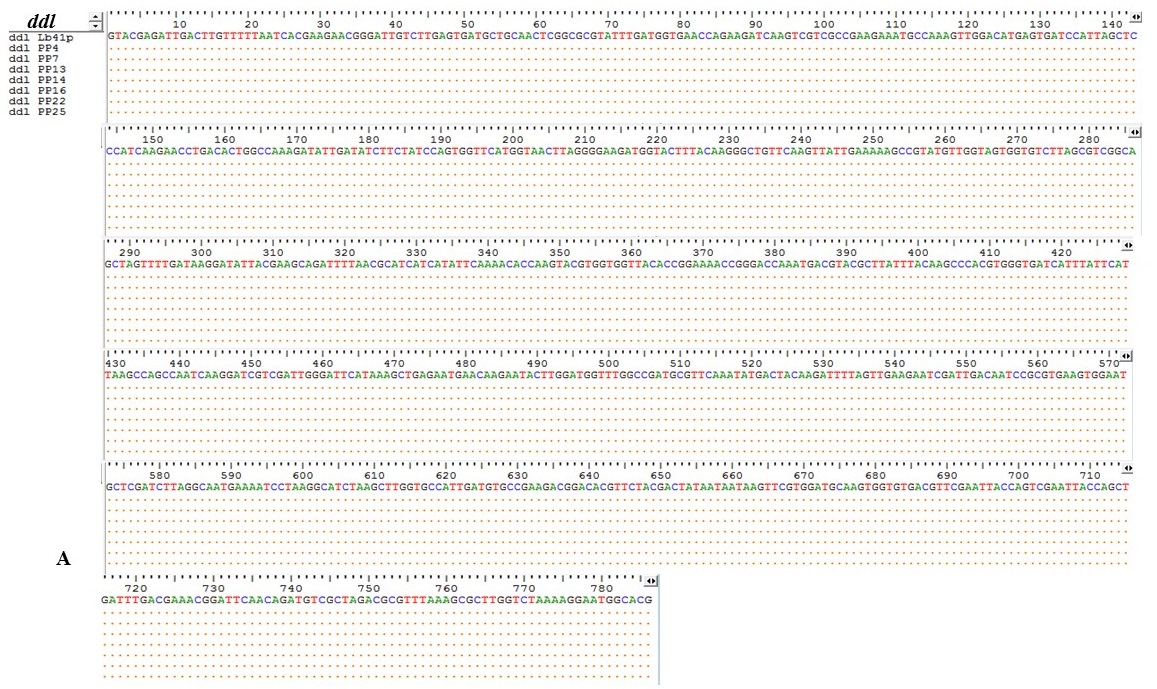


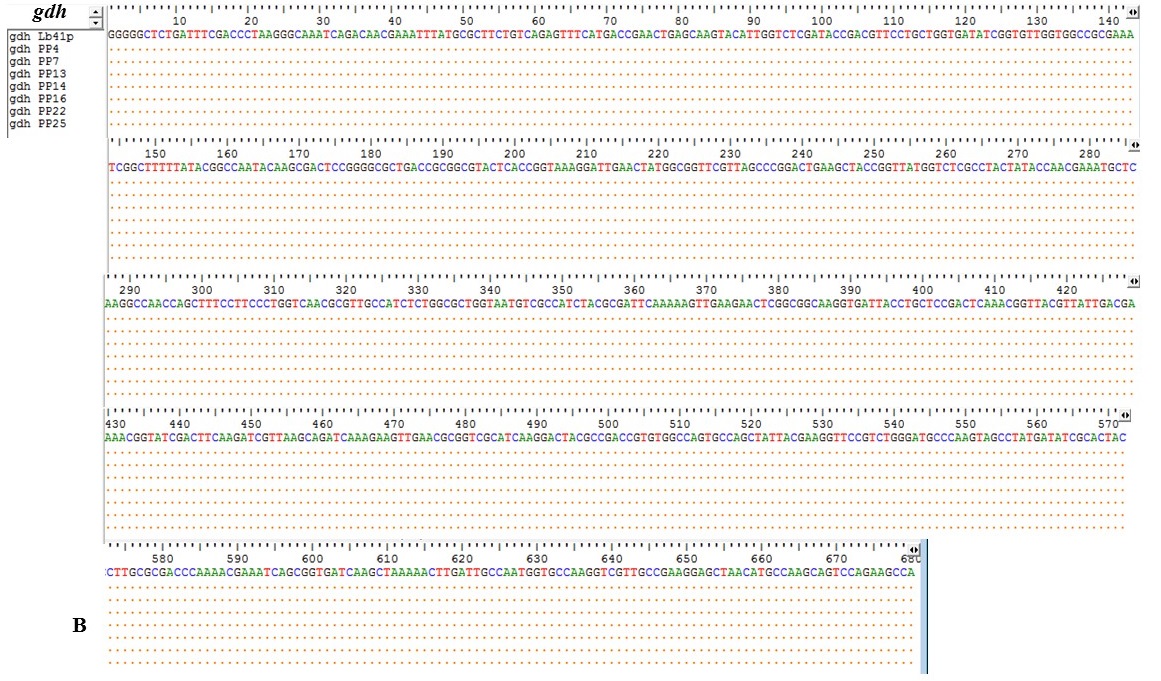


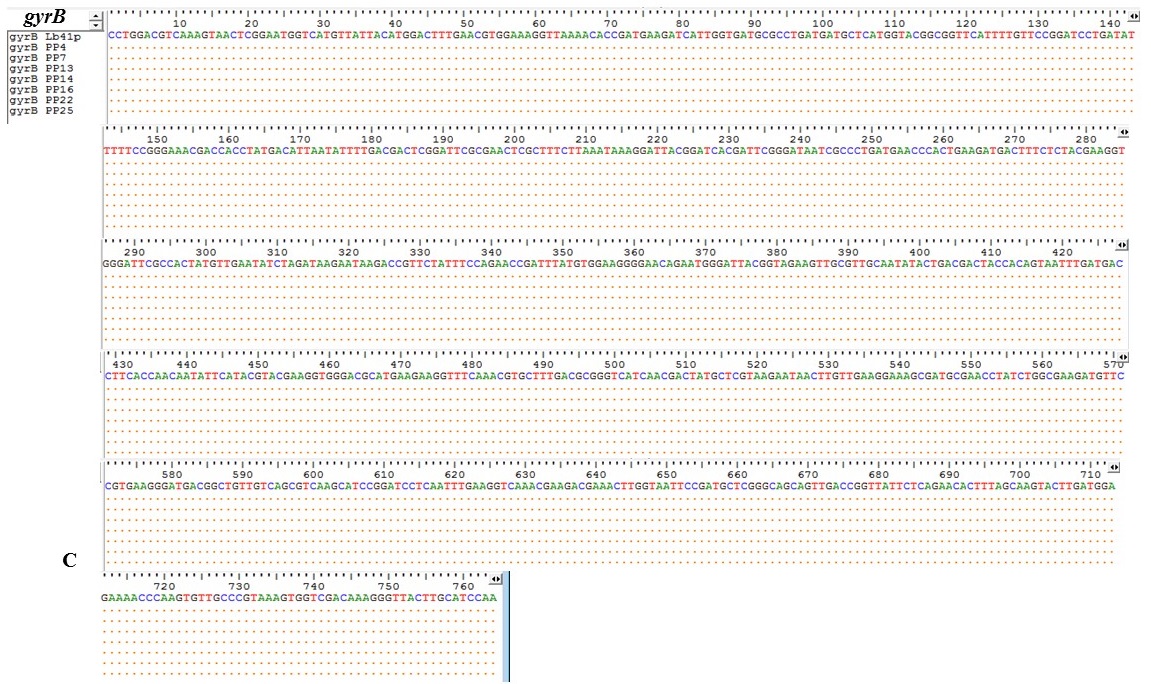


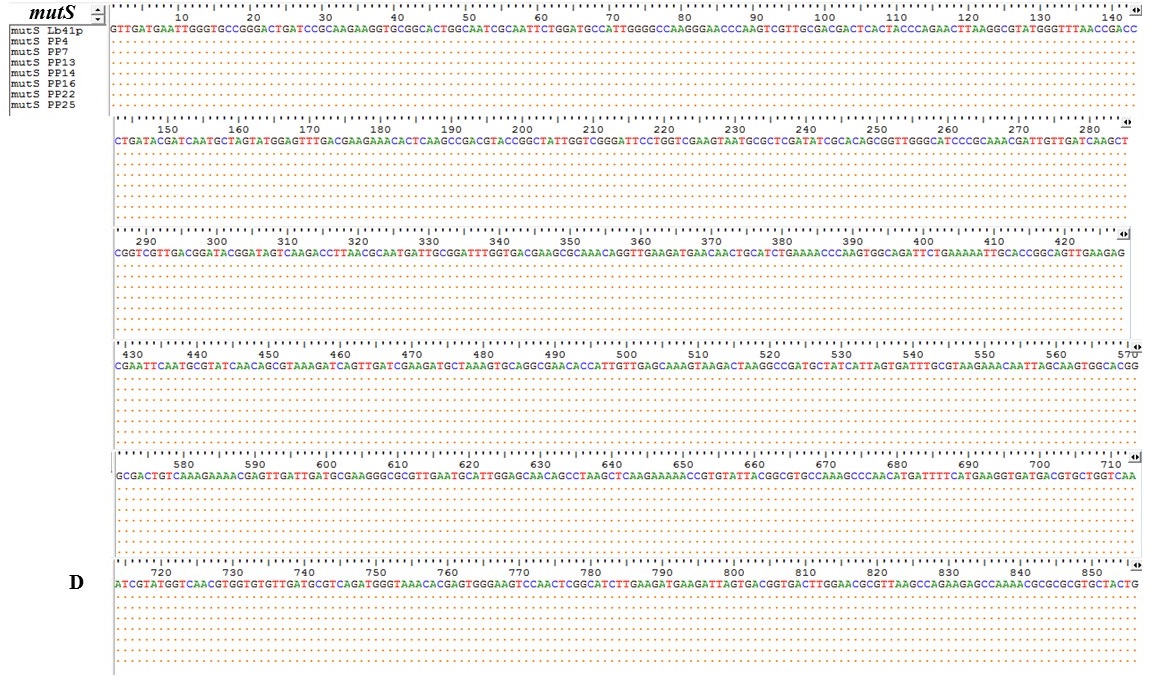


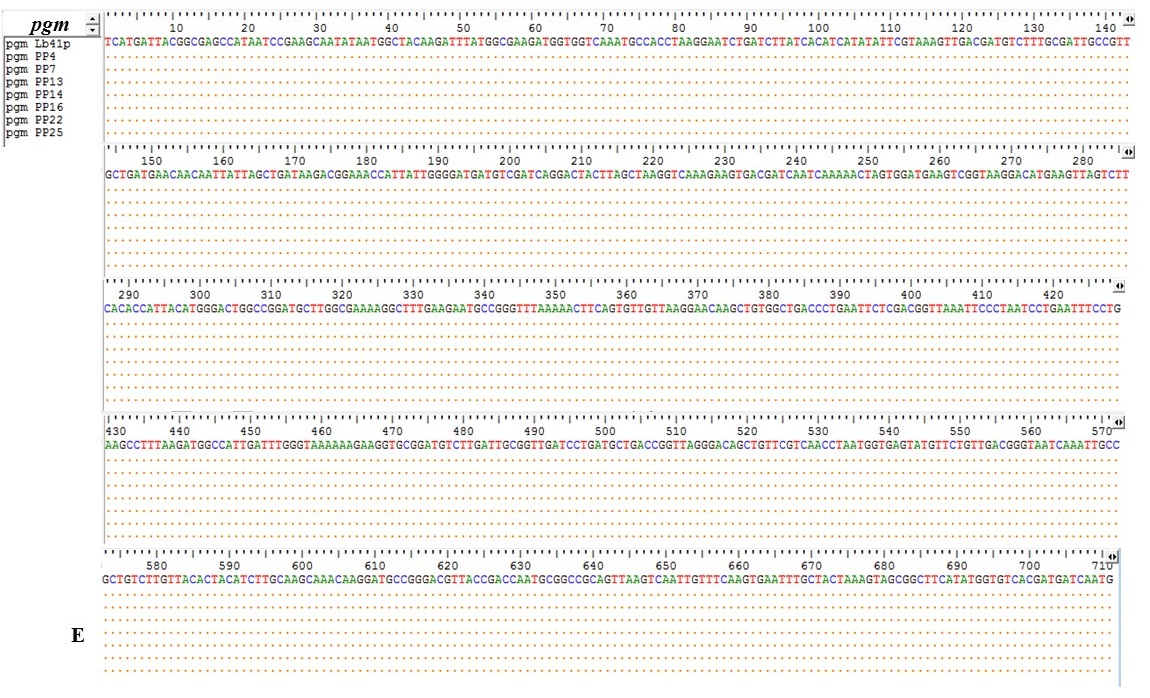


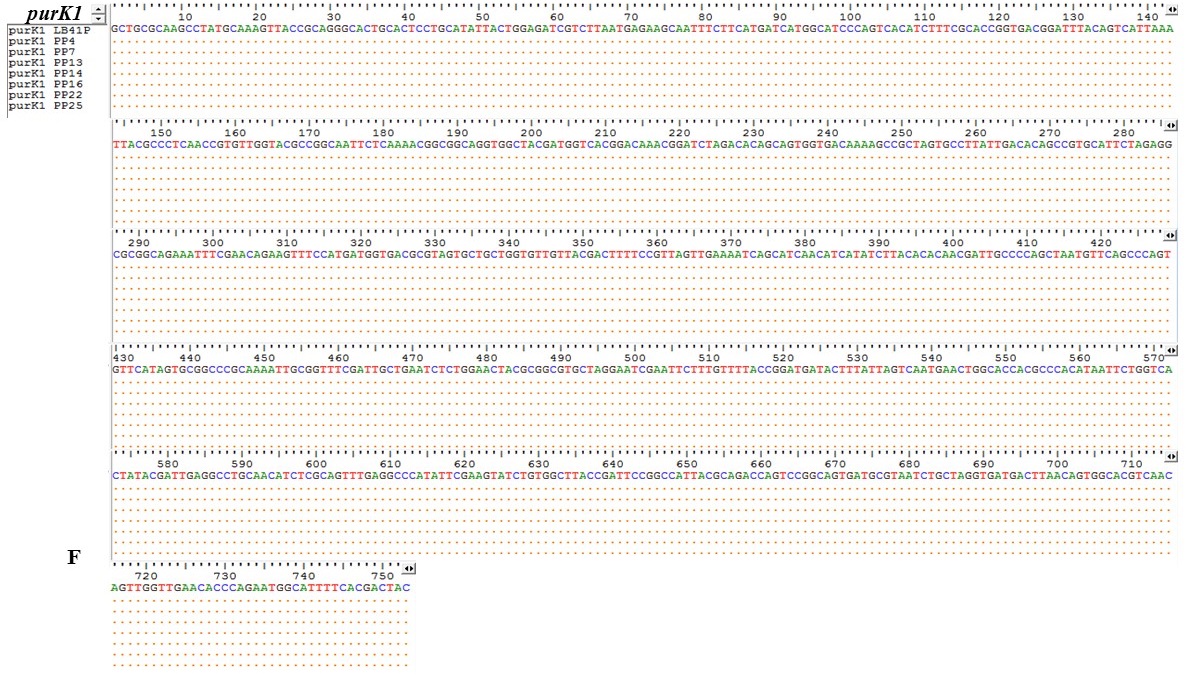


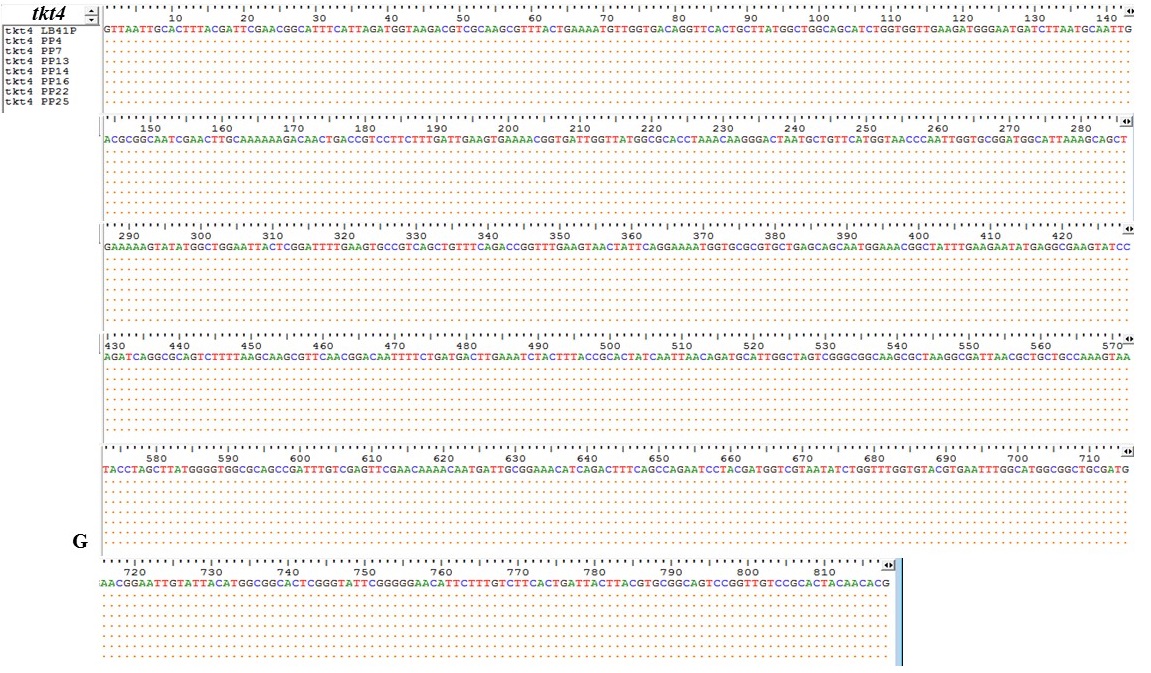

Supplement: Supplementary file 1 [file microorganisms-08-00005-s001.zip › Supplemenray figures Microrganisms.docx]
