# Supplementary material for: Tracking of Intentionally Inoculated Lactic Acid Bacteria Strains in Yogurt and Probiotic Powder
Source: Microorganisms. 2019 Dec 18;8(1):5. doi: 10.3390/microorganisms8010005 (PMC7022703; doi:10.3390/microorganisms8010005)
Supplement: Supplementary file 1 [file microorganisms-08-00005-s001.zip › Supplemenray Tables Microorganisms.docx]

Tracking of intentionally inoculated lactic acid bacteria strains in yogurt and probiotic powder

Anshul Sharma^1,2,3^, Jasmine Kaur^1^, Sulhee Lee^1^, and Young-Seo Park^1^**^*^**

1. Department of Food Science and Biotechnology, Gachon University, Gyeonggi-do 13120, Republic of Korea

2. Department of Food and Nutrition, Gachon University, Gyeonggi-do 13120, Republic of Korea

3. Faculty of Applied Sciences and Biotechnology, Shoolini University of Biotechnology and Management Sciences, Bajhol, Solan, Himachal Pradesh 173229, India

**^*^**Corresponding author

E-mail address: ypark@gachon.ac.kr (Prof. Y.-S. Park)

Mobile: +821088675378

**Supplemental Tables**

**Table S1**

PCR conditions used for Rep-PCR and for the amplification of housekeeping genes of the target colonies.

| **Primer*** | **Pre-denaturation** | **Denaturation** | **Annealing** | **Extension** | **No. of cycles** | **Post extension** |
| --- | --- | --- | --- | --- | --- | --- |
| (GTG)_5_ | 95°C/7 min | 95°C/2 min | 36°C/2 min | 72°C/2 min | 4 | 72°C/5 min |
|  |  | 95°C/1 min | 50°C/1 min | 72°C/1 min | 30 |  |
| REP | 95°C/7 min | 95°C/1 min | 41°C/1 min | 65°C/ 3 min | 35 | 65°C/16 min |
| ERIC | 94°C /3 min | 94°C/30 sec | 52°C/1.30 min | 68°C/8 min | 35 | 68°C/8 min |
| Z* | 94°C/2 min | 95°C/20 sec | (Av.Tm+1)°C/ 30 sec | 72°C/30 sec | 35 | 72°C/7 min |

***** REP- repetitive extragenic palindromic, ERIC-enterobacterial repetitive intergenic consensus, Z-protocol for amplification of housekeeping gene loci.

**Table S2**

Information of housekeeping loci and primers used for *Leu. mesenteroides* 11251 strain [1].

| **Gene** | **PCR primers (5′-3′)** | **Protein** | **Length (bp)** | **Sequence**  **Length (bp)** | **Annealing temp (°C)** |
| --- | --- | --- | --- | --- | --- |
| *groEL* | CCTGGCATACCACCTTGTG AAAATATGGGCGCTAAGCTTGT | Chaperonin GroEL | 1414 | 670 | 60 |
| *gyrB* | TTGGTGCTTCTGTTGTT CATCCCCAAAGCCAG | Gyrase subunit B | 1100 | 839 | 49.2 |
| *atpA* | CTTACGGCATGGCCC CAACATTGGTGCTGGT | ATP synthase subunit alpha | 530 | 496 | 51 |
| *pyrG* | AGCAACGTTCTCTGGTG CTGTAGCACAATTACGTTC | CTP synthase | 437 | 409 | 53.7 |
| *pheS* | ATTGAAGATCTTACGGC TCAACATAGCAAATCG | Phenylalanyl-tRNA  synthetase subunit alpha | 960 | 843 | 46.3 |
| *rpoA* | TTTGAAAAGCCAAATATTCAT GTTACGCACTTTCATCAT | RNA polymerase | 860 | 592 | 50.5 |
| *uvrC* | CGCACAGGAATTCTAACTGGA  TGCAGCATCGTCATTGGAA | Excinuclease ABC, subunit C | 366 | 360 | 58.3 |

**Table S3**

Information of housekeeping loci and primers used for *L. brevis* B151 strain [2].

| **Gene** | **PCR primers (5′-3′)** | **Protein** | **Length (bp)** | **Target**  **Sequence (bp)** | **Annealing temp. (°C)** |
| --- | --- | --- | --- | --- | --- |
| *gyrB* | AAGGCGTCAAGATTACCCTAAC  TTCATTTTTCATGATGTCGGGC | DNA gyrase  subunit B | 815 | 635 | 60.3 |
| *groEL* | GGATTGAAAAGGCGACTGG  TCATCAGCGATGATTAAGAGT | Chaperonin  GroEL | 400 | 374 | 57.4 |
| *pheS* | CAAGGAAATTTTGATGCGGA  GTTACAACCCTTTCCACCAC | Phenylalanyl  tRNA synthetase | 340 | 338 | 57.3 |
| *rpoB* | GATACCGGTGAAATTATTGCC  TCAATATCATCAGTGTTACCAA | RNA polymerase beta subunit | 314 | 215 | 57.0 |
| *dnaK* | TCGTCGTCGTTAACTTCGTG  CACATGGGTGAAGCTGGCTA | Chaperone  hsp70 | 1637 | 700 | 60.45 |
| *rpoA* | AGCTGTTACCAGCATTCAAA  GCTCTTCGATGGTCATCTC | RNA polymerase alpha subunit | 626 | 558 | 56.8 |
| *recA* | CACTTGACGTTGCCT  CCGTGCCATCTTTAATCT | Recombinase A | 570 | 453 | 49.8 |

**Table S4**

Information of housekeeping loci and primers used for *L. plantarum* LB41 strains [3].

| **Gene** | **PCR primers (5′-3′)** | **Protein** | **Length (bp)** | **Sequence length(bp)**  **LB41^K^** | **Sequence**  **length (bp)**  **LB41^P^** | **Annealing temp. (°C)** |
| --- | --- | --- | --- | --- | --- | --- |
| *ddl* | AACATGATGTTTCGAAGCG  GTTAGTAAAACCAGGTAACG | D-Alanine-D-alanine ligase | 916 | 818 | 786 | 53.0 |
| *gdh* | CCTTACAAGGGCGGCTTACG  ACGCCACCAGCATTGGCAGC | Glutamate  dehydrogenase | 859 | 790 | 680 | 63.9 |
| *gyrB* | GTGGTCTTCACGGGGTCG  TTCGACAATGAACAACAC | DNA gyrase  subunit B | 946 | 810 | 764 | 55.5 |
| *mutS* | AAGTACGTTCTCATCCCATATG  ATAACGCACACCCCGCAGGTC | DNA mismatch  repair protein | 987 | 872 | 856 | 60.4 |
| *pgm* | CTTGCGGCCAACCCCAGAAC  CCGTAGGATTCTTCAAAACC | Phosphoglucomutase | 863 | 772 | 711 | 59.2 |
| *Purk1* | TGACCTACGAGTTTGAAAAC  GGTGACATGACCCATCTTGCG | Phosphoribosylami-noimidazole  carboxylase, ATPase subunit | 838 | 761 | 753 | 57.6 |
| *tkt4* | GGTGATGGCGACTTAATGG  CCCATCCTCGCCGACCGC | Transketolase | 941 | 842 | 818 | 60.8 |

**References**

1. Sharma, A.; Kaur, J.; Lee, S.; Park, Y.-S. Genetic diversity analysis of *Leuconostoc mesenteroides* from Korean vegetables and food products by multilocus sequence typing. *Appl. Microbiol. Biot.* **2018**, *102*, 4853-4861.

2. Sharma, A.; Kaur, J.; Lee, S.; Park, Y.-S. Molecular discrimination of *Lactobacillus brevis* strains isolated from food products in South Korea using multilocus sequence typing. *LWT-Food Sci. Technol.* **2017**, *86*, 337-343.

3. de las Rivas, B.; Marcobal, Á.; Muñoz, R. Development of a multilocus sequence typing method for analysis of *Lactobacillus plantarum* strains. *Microbiology* **2006**, *152*, 85-93.
